# Ambiguous Loss Among Aging Migrants: A Concept Analysis- and Nursing Care-Oriented Model

**DOI:** 10.3390/healthcare13202606

**Published:** 2025-10-16

**Authors:** Areej AL-Hamad, Yasin M. Yasin, Lujain Yasin, Andy Zhang, Sarah Ahmed

**Affiliations:** 1 Daphne Cockwell School of Nursing, Faculty of Community Services, Toronto Metropolitan University, Toronto, ON M5B-2K3, Canada; lujain.yasin@torontomu.ca (L.Y.); sarah3.ahmed@torontomu.ca (S.A.); 2 Faculty of Nursing, University of New Brunswick, Fredericton, NB E3B-5A3, Canada; yasin.yasin@unb.ca

**Keywords:** ambiguous loss, aging migrants, concept analysis, transnational bereavement

## Abstract

**Introduction:** Ambiguous loss is a profound yet underexplored phenomenon in the lives of aging migrants. Older adults who have experienced migration often face disruptions to their sense of belonging, identity, and continuity across borders. These losses are compounded by aging, health challenges, and social isolation. Despite its significance, ambiguous loss among aging migrants has not been conceptually analyzed in depth, limiting the development of culturally responsive care practices. **Aim:** This concept analysis aimed to identify the defining attributes of ambiguous loss among aging migrants and to develop a conceptual definition that enhances our understanding of the phenomenon and informs future research and practice. **Method:** Walker and Avant’s eight-step concept analysis framework was applied to examine the concept of ambiguous loss in the context of aging migrants. A systematic keyword search was conducted across four databases (CINAHL, Medline, SCOPUS, PsycINFO), Google Scholar, and relevant gray literature, covering the years of 2010–2024. Covidence software supported the screening process. From 367 records identified, 146 underwent full-text review, and 74 met inclusion criteria. The analysis drew on literature synthesis, case exemplars, antecedents, consequences, and empirical referents. This review followed PRISMA (2020) reporting guidelines. **Results:** Four defining attributes of ambiguous loss among aging migrants were identified: (a) physical, social, and emotional loss; (b) displacement and loss of homeland; (c) erosion of social identity and agency; and (d) cultural and transnational bereavement. A conceptual definition emerged, describing ambiguous loss as a multifaceted experience of disconnection, intensified by aging, illness, economic hardship, and social isolation. The analysis also highlighted antecedents such as forced migration and health decline, as well as consequences including diminished well-being, resilience challenges, and barriers to integration. **Conclusions:** Ambiguous loss among aging migrants is a complex construct encompassing intertwined physical, social, and cultural dimensions of loss. This conceptual clarity provides a foundation for developing culturally responsive care models that promote adaptation, resilience, and social inclusion among older migrants.

## 1. Introduction

By mid-2020, an estimated 34.3 million migrants aged 65+ made up 12.2% of the global migrant population, with their numbers increasing by nearly 16 million in different countries [1]. Aging migrants are the fastest growing segment of the elderly population in Europe and North America [2]. An aging migrant is a foreign-born older adult who migrated in late life at age 65 or near retirement—whether through family reunification or forced displacement—and is now experiencing retirement while displaced in the host country [1]. In the past decade, over two-thirds of migrant deaths remain unidentified, leaving families and communities enduring uncertainty and ambiguous loss [3]. As migration rates rise and the proportion of aging migrants grows, ambiguous loss has become a key focus in understanding the psychological and social well-being of older adults [4]. Ambiguous loss is a profound psychological experience characterized by uncertainty and lack of closure regarding relationships, identity, and belonging [5,6]. Unlike concrete losses, such as the death of a loved one, ambiguous loss lacks closure and resolution [7], making it difficult for migrants to process and adapt. This phenomenon is especially relevant to aging migrants, who face prolonged separation from their homeland and family, as well as psychological disconnection due to cultural transitions and acculturation challenges [8]. Ambiguous loss in aging migrants is significant due to the psychological and emotional challenges of permanent uncertainty, unfinished loss, and the lack of closure [4,7]. Ambiguous loss in aging migrants extends beyond physical loss to include identity and belonging, as migration leads to the gradual loss of their original sense of self, traditions, and autonomy [8,9]. According to Perez and Arnold-Berkovits [10]), ambiguous loss may be associated with post immigration stress that is more intense for those of forced displacement due to sickness, aging, or financial instability.

Ambiguous loss arises when a person disappears or is psychologically absent, producing enduring uncertainty with psychological, social, economic, and administrative consequences. In the Mediterranean migration crisis, this affects families who remain behind or await relatives’ arrival [11]. For instance, families experience prolonged ambiguous loss—loved ones absent yet psychologically present—moving from anticipation to disappearance, searching, and chronic unknowing [12]. In Arizona and Sonora, this uncertainty triggers headaches, insomnia, anxiety, depression, and chronic disease, underscoring the need for system-level, culturally responsive support [12].

Unresolved losses associated with changes in family grieving process [13] and migration elevate the risks of depression, loneliness, and identity dissonance among aging migrants, affecting their overall quality of life and integration into host societies [2,14]. Ambiguous loss includes two main types impacting migrants’ well-being. Physical ambiguous loss occurs when someone is physically absent but psychologically present, such as older parents separated from their children by migration [7]. This type of loss is common among migrants unable to return to their homeland due to political, economic, or personal reasons, resulting in lasting emotional distress and a longing for reunification [8,10,15]. The second, psychological ambiguous loss, manifests when an individual is physically present but emotionally or cognitively absent, as commonly seen in aging migrants experiencing dementia, mental illness, or the erosion of cultural identity [16]. Many aging migrants experience both forms of ambiguous loss simultaneously, compounding their distress, complicating their adaptation to new environments, and challenging their sense of belonging [8,9,16].

Coping strategies for ambiguous loss among aging migrants encompass psychological adaptation, social support, and meaning making [16,17]. Studies indicate that transnational communication, maintaining strong ethnic community ties, and engaging in cultural or religious practices serve as protective factors against emotional distress [9,10,18]. For many aging migrants, religious beliefs and practices provide a source of continuity and resilience, reinforcing a sense of belonging and stability amidst the uncertainties of migration [19]. Additionally, the concept of both/and thinking, where individuals acknowledge both loss and presence; has been associated with greater emotional stability and adaptive coping [17]. Maladaptive coping mechanisms, like avoidance, emotional suppression, or idealizing the homeland, can result in prolonged grief, identity fragmentation, and difficulty integrating into host communities [18,20]. Interventions for ambiguous loss focus on cognitive reframing, community support, and culturally tailored mental health services [17]. Therapy models based on ambiguous loss theory emphasize increasing tolerance for uncertainty, recognizing that closure may not be possible [7]. Group therapies have been effective in validating experiences and fostering shared understanding, reducing isolation, and building resilience [21,22]. Language acquisition programs promote psychological well-being by enhancing social integration and access to healthcare [20,23].

Healthcare providers, particularly nurses, play a pivotal role in addressing the holistic care needs including emotional and psychological needs of aging migrants experiencing ambiguous loss [24]. Nurses can support family reunification, strengthen social networks, and implement psychoeducational programs to help aging migrants navigate the complexities of ambiguous loss [2]. Transcultural nursing care, which incorporates migrants’ beliefs, languages, and social norms, is instrumental in delivering effective and culturally competent interventions [10]. Training healthcare providers in cultural competence ensures care strategies meet the unique needs of migrant populations, improving their healthcare access and overall psychological well-being [20].

To translate this concept into practice, we position nursing as the point-of-care interface where ambiguous loss is most visible and actionable among aging migrants (home care, primary/community care, long-term care). Drawing on relational, culturally safe, grief- and trauma-informed principles, nursing operationalizes the concept’s antecedents, attributes, and consequences within interprofessional, community-anchored systems [16,25]. This orientation clarifies how assessment, communication, and pathway coordination can mitigate boundary ambiguity, disenfranchised grief, and social isolation.

As ambiguous loss continues to shape the lived experiences of aging migrants, further research is needed to explore its intersections with mental health, migration, social integration, and nursing care [26,27,28]. Existing literature highlights the importance of resilience, community belonging, and culturally sensitive interventions [2,17,29], yet gaps remain in understanding how healthcare systems can better support aging migrants experiencing ambiguous loss. This analysis will inform nursing interventions designed to enhance the well-being of aging migrants, contributing to the development of culturally sensitive and evidence-based nursing practices. This paper aims to (1) analyze and conceptually define ambiguous loss among aging migrants and (2) develop a conceptual model that links ambiguous loss among aging migrants with nursing care for older migrants.

## 2. Materials & Method

**Figure 1:**
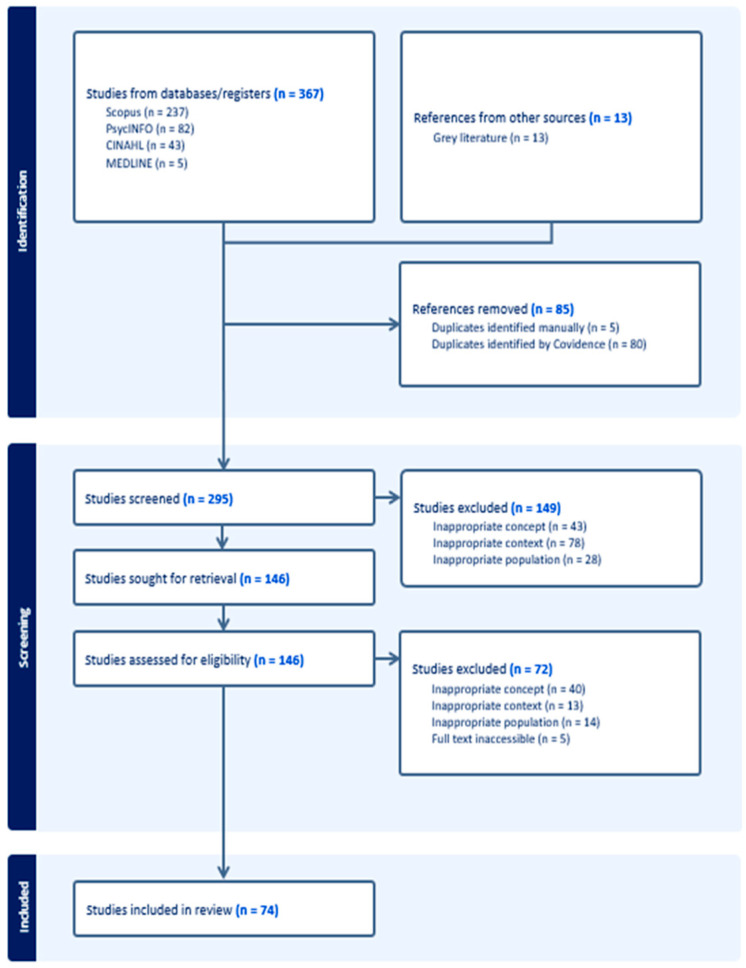
PRISMA flow diagram.

Our analysis applied the Walker and Avant [30] concept analysis framework, which is widely applied in nursing to systematically clarify underdeveloped concepts. This framework was selected because of its structured eight-step process, which strengthens theoretical precision and facilitates translation into nursing practice [30]. These steps include (1) identifying the concept of interest, (2) establishing the purpose of the analysis, (3) examining various uses of the concept, (4) outlining its defining characteristics, (5) constructing model cases that exemplify the concept, (6) identifying borderline and contradictory cases, (7) exploring antecedents and consequences, and (8) determining empirical indicators. This concept analysis adheres to the PRISMA (2020) reporting guidelines for systematic reviews. We applied Walker and Avant’s [30] concept analysis as the main methodological framework. PRISMA/PRISMA-ScR elements were used only to document and report the search and selection process (Figure 1), not as a study design. We conducted the concept analysis following Walker and Avant’s framework and interpreted the resulting attributes/antecedents through a nursing care lens to outline system-embedded assessment and intervention touchpoints [30].

### 2.1. Identifying the Concept and Search Strategy

The analysis began by defining key terms using various dictionaries (see Table 1), followed by a review of literature on “ambiguous loss,” “older adults,” and “migration.” A comprehensive search was conducted across multiple databases, developed in collaboration with a research librarian. The search used broad keywords, refined with MeSH terms and adapted for each database using Boolean operators and wildcards. To ensure accuracy, the strategy was reviewed by a second librarian following PRESS guidelines [31]. The finalized search strategy is detailed in (Appendix A). A cross-disciplinary uses (nursing, gerontology, migration studies, psychology, anthropology, social work) was conducted to ensure a comprehensive search and to prevent construct overlap and to delineate the boundaries of ambiguous loss. See Table 1 for the defining and distinguishing related constructs including migratory loss and grief and transnational bereavement.

### 2.2. Screening Process

The review identified 367 records from databases including CINAHL (*n* = 43), Medline (*n* = 5), SCOPUS (*n* = 237), PsycINFO (*n* = 82), and Google Scholar (*n* = 13), limited to publications from 2010 to 2024. After removing 85 duplicates and incomplete entries, 295 records were screened. Three reviewers (LY, SA, AZ) independently assessed titles and abstracts, with discrepancies resolved by discussion or a third reviewer (AA). Of these, 149 were excluded, and 146 full-text articles were reviewed. Following eligibility assessment, 72 were excluded, resulting in 74 studies included in the final review. No additional sources were identified through reference list checks. Eligibility criteria were: (a) population/focus on aging migrants and/or their caregivers/providers addressing ambiguous loss in later life; (b) concept relevance defined as explicit discussion of ambiguous loss or closely related constructs (see Table 1) linked to aging/migration; (c) host-country health, social, or community contexts; and (d) empirical or theoretical/conceptual peer-reviewed articles (editorials/letters excluded unless providing definitional clarity). Records were limited to English due to team language fluency and lack of translation funds; the date range was unrestricted with an emphasis on recent literature. We followed the PRISMA-ScR guidelines [36] to ensure a systematic and transparent literature review, supporting rigorous record selection, synthesis, and strengthening the concept’s theoretical foundation and relevance to practice. The PRISMA flow diagram (Figure 1) illustrates the entire search and selection process.

### 2.3. Analysis

Titles and abstracts were first screened for eligibility, followed by full-text reviews based on inclusion criteria. Using inductive content analysis [37]. Three reviewers (LY, SA, AZ) independently coded all included sources using inductive content analysis in NVivo. A shared codebook—containing operational definitions, inclusion/exclusion notes, and exemplar excerpts—was developed and iteratively refined. Coding disagreements were resolved by consensus, with a third reviewer (YY) adjudicating unresolved items, and an audit trail of decisions and analytic memos was maintained. We also included a brief reflexivity statement to acknowledge team positionality and steps taken to minimize bias. The findings were organized into attributes, antecedents, consequences, and empirical referents. Model and supporting cases were developed from literature these cases were derived from published literature and synthesized to exemplify defining attributes and boundaries and authors’ practice experience served only for ecological plausibility checks. Ethical approval was not required.

Our team includes researchers with prior work in migration, aging, and bereavement. We recognize that our practice-oriented lens could foreground care-system solutions and nursing perspectives. To minimize bias, we bracketed assumptions through independent double-coding, iterative codebook development, consensus adjudication with third-reviewer input, and an audit trail of analytic decisions/memos. We also triangulated definitions across disciplines and explicitly distinguished related constructs to reduce construct overlap.

## 3. Results

### 3.1. Term Definitions and Relevant Concepts

To understand “ambiguous loss among aging migrants,” it is useful to define key terms and examine related concepts. The literature reveals varied terminology describing similar experiences. This review clarifies the term aging migrant and explores related concepts, migratory loss and grief and transnational bereavement, highlighting their theoretical distinctions and evolution over time (see Table 1).

### 3.2. Defining Attributes

According to Walker and Avant [30], attributes represent the fundamental traits that define a concept, making it distinguishable from related ideas.

**Physical, Social and Emotional Loss**: Physical loss among older migrants includes the loss of possessions [38], land [39], housing and financial resources [40,41], increasing reliance on social capital [38]. Social loss involves disruptions in networks, such as losing a spouse [42,43], family [44,45], friends [46], caregivers [47], and employers [48], often due to death [49,50] or migration [51]. Older migrant women may feel loneliness after losing male authority figures [39,52]. Emotional loss accompanies these physical and social losses, including declining self-confidence [44] regret after bereavement [53], and emotional effects of childhood separations, such as rootlessness and guilt [54].

**Loss of Homeland and Displacement:** Loss of homeland due to forced displacement often results from threats to personal safety, including war, conflict, violence, and political persecution [4,38,39,45,55]. Contributing factors include regime changes, government corruption [39], and environmental crises such as climate change, pollution, and natural disasters [56]. Displacement leads to cultural losses, including loss of heritage, shared culture, and language [4,45], often resulting in migratory grief [57]. In response, migrants remake a sense of home in host countries through cultural practices and community engagement [4].

**Loss of Social Identity and Agency:** Migration often leads to the loss of social roles for older migrants, causing devaluation and disempowerment, especially among men who lose leadership roles [45,52,58,59]. Many adopt nontraditional roles, but culture shock and lifestyle changes disrupt identity [46,60]. An inability to continue traditional practices, like farming, further challenges identity [60]. Loss of home ownership and independence, as seen in older Chinese migrants, reduces autonomy [40]. Older African migrants also struggle with identity renegotiation due to forced displacement [61]. Discrimination, ageism, and declining health further impact social identity and increase reliance on family support [42,47,62].

**Cultural and Transnational Bereavement:** Cultural bereavement involves grieving the loss of social structures, identity, and cultural practices due to being uprooted from one’s homeland [60]. Korean and Egyptian migrants, for example, experience the erosion of cultural identity and traditions during acculturation [39,57]. This grief is often tied to cultural trauma and fears about losing language, religion, and customs across generations [60]. Transnational bereavement occurs when migrants grieve distant losses without traditional mourning rituals, leading to disenfranchised grief, guilt, and isolation [49,63]. Migrants may rely on digital communication for transnational caregiving, while children and kin often take on roles as mediators of care and mourning across borders [38,48,63].

### 3.3. Identifying Model, Borderline and Contrary Cases

In concept analysis, Walker & Avant [30] emphasize the use of illustrative cases to clarify meaning and boundaries of a concept. A model case represents the concept in its most complete form, demonstrating all of its defining attributes and showing readers exactly what the phenomenon looks like in practice [30]. In contrast, a borderline case includes only some of the defining attributes, which helps highlight situations that share partial features of the concept but do not fully represent it [30]. Finally, a contrary case contains none of the defining attributes and therefore illustrates what the concept is not [30]. Together, these cases function as practical tools for sharpening conceptual clarity, allowing scholars and practitioners to distinguish the essential elements of a phenomenon from its partial or unrelated forms. To illustrate the concept of ambiguous loss among aging migrants, the authors constructed model, borderline, and contrary cases, as presented in (Table 2).

### 3.4. Antecedents

According to Walker and Avant [30] concept analysis method, *antecedents* are the prior events or conditions that must be in place for a concept to develop or take form. The following discusses the antecedents of ambiguous loss among aging migrants:

**Physical Aging and Frailty:** Physical aging among older migrants involves losses such as sensory decline [42], memory loss from dementia [64,65], hearing loss [66], immobility [47], and disability [59,67]. Frailty, chronic illness, and cognitive decline reduce independence and self-identity [10,20,44,68]. These issues often stem from past hardships like war, forced displacement, and limited healthcare access [61,69]. Cognitive decline leads to psychological absence, affecting familial relationships and emotional well-being [20,60]. Language attrition further isolates older migrants, deepening ambiguous loss and disconnect from both past and present identities [62,70,71,72].

**Economic Strain and Detachment:** Aging migrants often face economic insecurity due to disrupted career paths, low-paying jobs, and limited pension eligibility [68,73,74]. These financial pressures increase dependence on family, leading to feelings of burdensomeness and reduced autonomy [18]. Economic hardship also limits access to healthcare, forcing reliance on community services that may lack cultural sensitivity [72,75]. Social isolation is intensified by language barriers, cultural differences, and the loss of support networks from the home country, resulting in emotional distress and a sense of detachment [45,76,77].

**Shifting Family Dynamics and Caregiving:** Older adults, respected for guiding younger generations, experience a loss of status and decision-making power due to migration, especially when children adopt Western caregiving models [8,18,70]. This shift can create intergenerational tension, leaving aging migrants feeling undervalued and disconnected [55,58]. As filial piety erodes, institutional care and reduced family support may follow [4,69]. Transnational caregiving also causes emotional distress from physical separation [63]. Older women, in particular, experience greater losses in status and autonomy due to changing gender roles [59]. These evolving caregiving structures often lead to cultural tensions between independence and familial duty, intensifying feelings of ambiguous loss [4,49].

**Changing Cultural, Aging and Grief Rituals:** Cultural adaptation poses significant challenges for aging migrants as they balance preserving their cultural identity with adapting to new societal norms [60,72]. This often leads to the gradual loss of traditional values, creating internal conflict, emotional distress, and identity struggles [61,62,71]. Difficulty adapting can increase anxiety and depression due to displacement and social exclusion [75,78]. Migration disrupts traditional mourning practices, preventing participation in meaningful rituals like visiting ancestral graves or holding funerals, deepening the sense of loss [4,49,50,79]. Aging migrants often report feeling unsupported due to the lack of culturally sensitive end-of-life care in host countries [63,72].

### 3.5. Consequences

According to Walker and Avant [30] concept analysis method, consequences are the outcomes or effects that follow the occurrence of the concept being analyzed, highlighting its results or implications. The following are the consequences of ambiguous loss among aging migrants:

**Strained Family Ties and Generational Tensions:** Weakened family ties and intergenerational tensions are common among aging migrants due to cultural shifts, language barriers, and migration-related separation. Transnational caregivers often feel guilt and distress over unmet caregiving expectations [2,8]. Older immigrants report strained relationships as younger generations adopt host country values [18,45,51]. Refugee elders face isolation and reversed caregiving roles, relying on younger relatives for daily support, which can lead to resentment and loss of autonomy [4,20,58,60]. Historical trauma among post-war migrants further disrupts intergenerational bonds where past experiences remain unspoken [39,54].

**Declined Health with Limited Care Access:** Older immigrants and refugees face declining physical and mental health, with limited care access, report poor social support and increased vulnerability [51], and elders with hearing loss struggle due to stigma and financial barriers [66]. Refugees experience PTSD and depression linked to language and financial issues [58]. Environmental and economic stressors, including COVID-19, have worsened mental health [56,80]. Loss of social networks from forced migration adds emotional strain [76]. Language barriers and inadequate healthcare contribute to loneliness and poor disease management [10,18,81].

**Increased Loneliness, Isolation and Grief:** Older immigrants with spousal bereavement, facing cultural and linguistic barriers that hinder emotional support, leading to prolonged grief and isolation [82]. Latino immigrants experience ambiguous loss of their homeland and contributing to social withdrawal [10]. Hmong older immigrants report loneliness due to loss of cultural identity and intergenerational disconnection [52]. Similarly, Muslim immigrants experience existential loneliness from losing religious and communal spaces [62]. Bhutanese refugee elders face cultural trauma and bereavement, intensifying isolation and distress [60]. These cumulative losses lead to avoidant behaviors, further reinforcing cycles of loneliness and grief [75,83].

**Heightened Displacement and Diminished Belonging:** Bhutanese refugee elders face cultural trauma in exile, leading to loss of identity and disconnection [60]. Cuban American exiles experience ambiguous loss of their homeland, deepening cultural fragmentation and nostalgia [10]. Older Italian migrants in Australia struggle with intergenerational family ties, reinforcing their outsider status [42]. Southeast Asian refugees in the U.S. feel distanced from their children due to acculturation gaps, intensifying familial tensions [58]. Forced migration strains social networks, as seen among Japanese immigrant widows navigating bereavement in cultural marginalization [50]. Syrian refugees experience ongoing mourning due to the inability to return home [4]. Hmong elders report loneliness from disrupted cultural continuity and intergenerational misunderstandings [52,55]. Mizrahi women in Tel Aviv reflect on their eroded cultural presence due to shifting communal structures [79]. These experiences highlight how displacement affects identity and deepens cultural grief [18,76].

### 3.6. Empirical Referents

Empirical referents are observable indicators that align with a concept’s defining attributes, helping to identify and measure its presence in practice as explained by Walker& Avant [30]. For aging migrants, empirical referents of “ambiguous loss” include: Perez and Arnold-Berkovits [10] categorize migrants based on their levels of ambiguous loss of homeland (ALH) and relative satisfaction (RS) with their host country, considering how nostalgia, homesickness, and socio-political factors influence their experiences. Segmented Assimilation theory by Poryes and Zhou Min [84] explains how migrants integrate into new societies at different rates, with some achieving upward mobility and inclusion, while others face systemic barriers and persistent uncertainty. The psychological impact of migration can also be understood through the Continuity Theory of Normal Aging [85] that suggests that individuals strive to maintain a consistent sense of self despite external disruptions, such as migration. Disengagement Theory [86] posits that aging individuals naturally withdraw from previous roles and relationships, a process heightened for older migrants who feel disconnected from both their homeland and host society. Stress and Coping Theories [87], explores how individuals manage stressful situations, including cultural displacement and legal precarity, and the emotional toll of migration. Additionally, FRAIL Scale [88] is a valuable tool for assessing physical and cognitive vulnerabilities. Psychosocial distress related to ambiguous loss can also be measured using the Social Screening Scale [89] which evaluates risk factors such as sadness, outside activity, cognition, income adequacy, attachment to neighbors, and lethargy.

### 3.7. Model Development

Having established the defining attributes, antecedents, and consequences of ambiguous loss among aging migrants, we now translate these elements into a conceptual model. The model specifies the pathways by which contextual moderators (e.g., migration trajectory, language access) shape the expression of attributes and the downstream consequences and identifies system touchpoints for assessment and support.

Drawing on the defining attributes outlined in the existing literature, ambiguous loss among aging migrants can be conceptualized as a multifaceted experience marked by physical, social, and emotional loss, displacement, diminished identity, and cultural bereavement. Rooted in aging-related frailty, chronic illness, and cognitive decline, it is intensified by economic hardship, social isolation, and shifting family roles. Struggles with cultural adaptation and changing mourning rituals deepen feelings of displacement, weakened identity, and loss of belonging, leading to loneliness, grief, avoidant behaviors, strained family ties, and declining health.

Having delineated the defining attributes (e.g., boundary ambiguity, chronic sorrow, ambivalence) and antecedents (e.g., separation, role disruption, legal/linguistic barriers), we map these elements to system touchpoints where nursing actions occur within interprofessional pathways: (a) language access and therapeutic communication, (b) navigation and continuity, (c) grief-/trauma-informed mental-health supports, and (d) community linkage and social prescribing. Where language discordance is an antecedent, nurses ensure routine use of professional interpreters, enabling accurate assessment of ambiguous loss and safer care [90]. When legal/organizational barriers contribute to antecedents (eligibility gaps, fragmented referrals), nurses coordinate “no-wrong-door” navigation and continuity with primary care and settlement services [25,91]. For attributes such as disenfranchised grief or hypervigilance, nurses initiate screening, brief interventions, and stepped-care referrals within interprofessional teams [16,25]. Where isolation sustains consequences, nurses connect clients to culturally meaningful groups and social-prescribing options, with attention to equity and evaluation [92].

**Figure 2:**
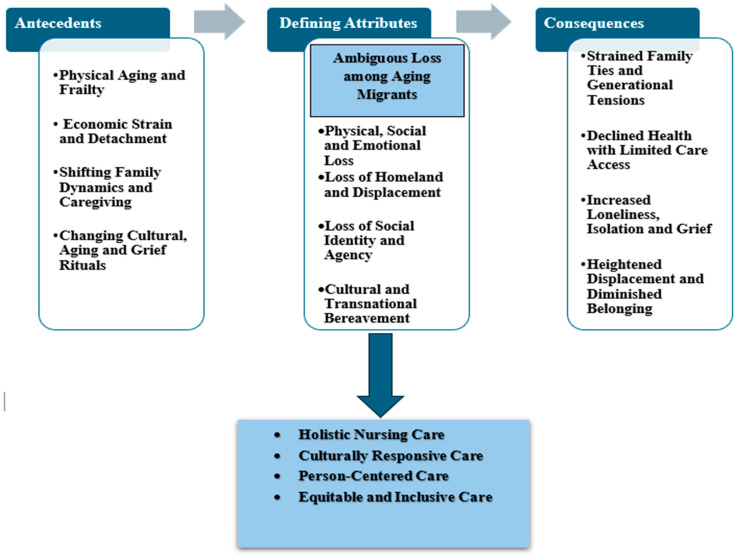
Conceptual model for ambiguous loss among aging migrants and nursing care for older migrants.

Having established the concept’s antecedents, attributes, and consequences, we synthesize these elements into a conceptual model that specifies pathways and system touchpoints, directly informed by and traceable to the analysis by Walker & Avant. A conceptual model for ambiguous loss among aging migrants and nursing care is proposed, illustrating its defining attributes, antecedents, consequences, and connection to core nursing principles (Figure 2). The model emphasizes the attributes at the heart of aging migrants’ experiences, framed by antecedents necessary for the concept’s emergence. The consequences of these attributes are then linked to the core principles of nursing care for older adults highlighting the importance of holistic, person-centered, culturally responsive, and inclusive care in addressing ambiguous loss.

## 4. Discussion

The aim of this concept analysis was to explore the defining attributes of ambiguous loss among aging migrants, develop a clear definition, and propose a model illustrating its factors and outcomes. Key attributes include physical, social, and emotional loss, loss of homeland, social identity, and cultural bereavement. As shown in the model (Figure 2), these attributes stem from antecedents like aging, economic strain, family dynamics, and shifting cultural practices. Consequences may include strained family ties, health decline, isolation, and diminished belonging. The model emphasizes holistic, person-centered, culturally responsive care, highlighting how nurses can address the emotional and social needs of aging migrants. This analysis draws on literature focused on ambiguous loss in aging and migration contexts. While the term is gaining recognition, definitions and applications remain inconsistent across studies [17,20]. This analysis synthesizes existing literature and aligns it with nursing practices for older adults, offering a clearer understanding of ambiguous loss among aging migrants. Further development can be achieved by linking the concept to nursing theories and practices, ensuring that care models address the diverse experiences and needs of aging migrants.

When applying this concept analysis to aging migrants, it is important to consider that the relevance of ambiguous loss may vary based on the nature of the loss [17]. Aging migrants experience unresolved grief over lost loved ones and homeland [93], traditions, oscillating between hope, mourning, and assimilation in their new society [11]. These losses are intensified by life’s discontinuity, making aging migrants more vulnerable as they idealize the past while trying to create a sense of home in a new environment [2]. Ambiguous loss, such as uncertainty around social security, war, or abduction, affects aging migrants with family in their homeland, leading to guilt and increased risk of depression and anxiety due to their inability to support loved ones [10]. The loss of agency, control over health, and inability to engage in traditional mourning rituals contribute to significant psychological distress for aging migrants [2]. Ambiguous loss, often a lifelong process [10], can have a significant psychological impact, leading to fatigue, depression, and exacerbating comorbidities among aging migrants especially those from diverse ethnic backgrounds [94]. Shen [18] highlights that processing ambiguous loss and migratory grief is complex for the elderly, as they may suppress pain to avoid feeling burdensome. Addressing this loss is crucial for social inclusion, mental well-being, and culturally adaptive coping, as grief varies across ethnicities [10]. Culturally safe communication and recognition of their experiences can help aging migrants navigate loss and, in some cases, reclaim their identities [11]. Aging migrants’ well-being is influenced by discriminatory policies and their control over their new lives and their grief is often dismissed, with expectations of assimilation [10].

Older migrants with limited English proficiency experience worse health outcomes, highlighting the need for nurses to communicate in their native language to improve care and well-being [8]. Identity loss can foster worthlessness and social isolation, particularly among older migrants, as it often goes unrecognized [18]. Aging migrants’ diminished sense of purpose, compounded by language barriers, dependency, family conflict, and the dual burden of hope and mourning, fosters silent grief and accelerates their decline [20]. Additionally, older migrants play a vital yet often overlooked role in society through unpaid household labor, lacking social identity or personal pursuits [95]. Their heightened risk for chronic illnesses and mental health challenges emphasizes the need for nurses to monitor their well-being and ensure proper support systems [94]. Social support is vital in easing suffering, restoring purpose, and helping aging migrants manage ambiguous loss by allowing them to release grief and personal burdens [18]. Mazzarelli et al. [11] emphasize the need for an ethno-psychological approach in nursing care, requiring cultural humility [96] to understand migrants’ mourning and coping within their specific contexts. By providing culturally sensitive care [96], mental health support and embracing an anti-ageist and anti-racist approach, nurses can assist aging migrants in achieving a sense of closure, peace and well-being [97].

### 4.1. Systemic Barriers and Health-System Responsibilities in Addressing Ambiguous Loss

Although our definition encompasses voluntary, family-reunification, and forced migration, ambiguous loss is not experienced uniformly. The migration trajectory modulates both antecedents and attributes. In conflict-related or forced displacement, antecedents more often include acute trauma, legal precarity, and prolonged separation with disrupted communication, attributes commonly present as boundary ambiguity, hypervigilance, and disenfranchised grief. In family reunification, antecedents tend to involve bureaucratic delays, role renegotiation, and transnational caregiving; attributes include ambivalence, role confusion, and intergenerational tension. In labor/education-driven migration, antecedents may include circular mobility and economic obligations; attributes often manifest as chronic homesickness and identity liminality amid relatively stable ties. These variations underscore the need to tailor assessment and support to sociocultural context and migration pathway.

Ambiguous loss among aging migrants rooted in separation from people/places and the uncertainty of return—manifests as boundary ambiguity, chronic sorrow, and disenfranchised grief [4,98]. Access to care is constrained by structural and informational barriers, including limited knowledge of entitlements, language discordance, eligibility gaps, cost, and mistrust, which delay help-seeking and intensify antecedents and attributes of ambiguous loss [91]. A system response is therefore required. Scholars argued that the WHO’s Integrated People-Centred Health Services framework supports community-anchored, coordinated models that embed proactive outreach with settlement/faith partners, “no-wrong-door” navigation, and co-located primary, mental-health, and social services [25]. Routine language access using professional interpreters improves safety, quality, and utilization [90]. Community-linking interventions such as social prescribing may help rebuild meaning and connection but should be implemented with equity safeguards and ongoing evaluation [92]. Within this larger effort, nursing roles include public/community health and Nurse Practitioner-led outreach and navigation; gerontological nursing for screening, caregiver support, and continuity; and mental-health/psychiatric nursing for grief- and trauma-informed care ideally integrated with community organizations and interpreter services.

### 4.2. Nursing Core Principles for Working with Older Migrants

Addressing ambiguous loss requires system-level redesign embedded interpretation, coordinated navigation, and community-partnered care so that nursing contributions (public/community health, gerontological, and mental-health nursing) operate within integrated pathways rather than isolated encounters [25,91]. Embedding these functions in routine practice clarifies role boundaries, improves access, and aligns with the concept’s emphasis on continuity of ties and meaning reconstruction [16].

Holistic, culturally responsive, person-centered, and equitable care approaches are essential in supporting aging migrants who experience ambiguous loss. Holistic nursing care acknowledges not only physical health but also the psychological, emotional, and cultural disruptions older migrants face due to displacement and identity shifts [24]. By integrating strategies like reminiscence therapy for loneliness [22], life review therapy [21], and spiritual support [99], nurses can help aging migrants find meaning in their experiences while promoting their resourcefulness [100] and continuity of identity [101]. Culturally responsive care complements this approach by recognizing the importance of cultural beliefs, languages, and practices in shaping how aging migrants’ express grief and cope with separation or dislocation [102]. When nurses incorporate culturally meaningful traditions such as religious practices, native language use, or community ties into care plans [29], they foster emotional safety and connection, while also reducing feelings of invisibility and cultural alienation [103]. In parallel, person-centered care emphasizes the individual’s narrative, values, and lived experiences [104]. For aging migrants, who often face fragmented family ties and shifting social roles [105], this approach respects their autonomy and acknowledges the depth of their loss [26]. Nurses can enhance engagement by tailoring care strategies to each individual’s cultural background, communication style, and emotional needs [106]. Equitable and inclusive care ensures that access to quality healthcare is not limited by age, migration status, language, or socioeconomic factors [107,108]. Many aging migrants face systemic barriers such as language inaccessibility, legal precarity, and discrimination [109,110], which exacerbate feelings of isolation and loss [111]. Nurses play a pivotal role in advocating for inclusive services such as interpreter support and culturally tailored programming [100] while affirming diverse expressions of grief [2]. Collectively, these interwoven nursing approaches offer compassionate, responsive and supportive frameworks [15] for addressing the complex emotional and cultural realities of ambiguous loss among aging migrants.

The developed model is tentative and intended to guide assessment and care, not to claim a fully validated theory. Future work should (1) qualitatively test pathway plausibility across migration trajectories and contexts; (2) develop/adapt empirical referents (e.g., boundary-ambiguity indicators) with psychometric evaluation; (3) conduct feasibility and mixed-methods studies of interpreter-enabled navigation, grief-informed groups, and role-clarification interventions; and (4) use longitudinal designs to examine changes in ambiguous loss attributes and consequences with targeted supports.

### 4.3. Implications

The findings of this concept analysis highlight the urgent need for tailored, culturally sensitive nursing models to support aging migrants experiencing ambiguous loss. Nurses working with older migrants must be equipped to address the complex interplay of psychological distress, cultural bereavement, and social isolation among this population [2,17]. Integrating holistic [24], person-centered [106,112,113], culturally responsive and equitable care [114] can promote emotional resilience and foster belonging [115]. Health systems should prioritize language access, intergenerational support strategies, and health services tailored to migrants’ diverse backgrounds [20]. Furthermore, training healthcare providers in cultural humility and trauma-informed approaches will strengthen inclusive care practices and mitigate identity loss and marginalization [10,18]. Nurses can use this model to enhance aging migrants’ well-being and address the long-term effects of ambiguous loss. Future research should focus on developing tailored care models that address the intersection of aging, migration and loss, [28], ensuring that healthcare services remain inclusive and responsive to the needs of aging migrant population.

The developed model is tentative and intended to guide assessment and care, not to claim a fully validated theory. Future work should: (1) qualitatively test pathway plausibility across migration trajectories and contexts; (2) develop/adapt empirical referents (e.g., boundary-ambiguity indicators) with psychometric evaluation; (3) conduct feasibility and mixed-methods studies of interpreter-enabled navigation, grief-informed groups, and role-clarification interventions; and (4) use longitudinal designs to examine changes in ambiguous loss attributes and consequences with targeted supports.

### 4.4. Limitations

This analysis is limited to English-language literature, potentially excluding non-English perspectives. Perceptions of ambiguous loss may vary by cultural background and migration experience, affecting the generalizability of findings. While the proposed model offers a useful framework, it requires further empirical testing. Future research should explore ambiguous loss in diverse cultural contexts to refine the model. Despite these limitations, this analysis provides valuable insights for advancing the field.

## 5. Conclusions

Ambiguous loss among aging migrants is shaped by physical, social, and emotional displacement, the loss of identity, and cultural bereavement. This analysis highlights the psychological and health impacts of unresolved grief, isolation, and changing family dynamics. Nurses play a vital role in addressing these effects through holistic, culturally responsive, and person-centered care. This concept analysis offers a clearer definition of ambiguous loss among aging migrants, identifies four defining attributes and key antecedents/consequences, and organizes these elements into a practice-oriented conceptual model. Centering AL—rather than a general catalog of losses—clarifies why boundary ambiguity, disenfranchised grief, liminality/chronic sorrow, and ambivalence co-occur under sustained uncertainty and where practice can intervene. By fostering inclusive healthcare, nurses can help aging migrants navigate loss, regain agency, and maintain a sense of belonging. In sum, a nursing-led, system-enabled approach grounded in language access, navigation, grief/trauma-informed care, and community linkage operationalizes the concept of ambiguous loss for aging migrants and strengthens equity-oriented practice.

## Figures and Tables

**Table 1:** Term definitions and relevant concepts.

Term	Definition
The meaning of each word from various dictionaries	A search of the Merriam-Webster Dictionary, American Heritage Dictionary, and Oxford English Dictionary yielded no results for the terms “ambiguous loss” and “aging migrants.” To refine our analysis, we searched for “ambiguous,” “loss,” “aging,” and “migrant” separately. While all three sources provided similar definitions, Merriam-Webster offered the most comprehensive definition of “ambiguous,” describing it as “doubtful or uncertain especially from obscurity or indistinctness” [32]. For “loss,” the Oxford English Dictionary provided the most detailed definition, describing it as “the fact of losing (something specified or contextually implied)” [33]. The American Heritage Dictionary offered the most precise definitions for “aging” and “migrant,” defining “aging” as “the process of growing old or maturing” and “migrant” as “a person who leaves one country to settle permanently in another: an immigrant” [34,35].
Migratory loss and grief	Migratory loss can include aspects of life that were lost during migration such as hobbies, lands and comforting locations [18]. The grief of leaving the graves of their loved ones or losing their hard-earned wealth, possessions or selling possessions along with the stressors of finding employment in a new country can be psychologically distressing [8].
Transnational bereavement	With migration, aging migrants might deal with major life events such as death, illness and significant life events which may trigger the feeling of transnational bereavement [2] and being left out [8].

**Table 2:** Model and additional cases of “ambiguous loss among aging migrants”.

Type & Definition	Case
Model case: A representative example that highlights every defining attribute of the concept [30].	Maryam, a 70-year-old Syrian refugee, grapples with profound physical, social, and emotional losses. Once a respected elder, she now feels invisible, mourning her homeland, late husband, and diminished role within her family. Language barriers and cultural differences isolate her, while the loss of familiar traditions and mourning rituals deepens her grief. Aging, chronic illness, and economic hardship further erode her independence. She avoids medical care out of fear of dependency and spends most days alone, disconnected from her family. Shifts in caregiving roles leave her feeling worthless and unseen. Trapped between a lost past and an unfamiliar present, Maryam lives in silent grief and quiet withdrawal.
Borderline case: An illustration of the concept capturing many, but not every, defining attribute [30].	Taher, a 65-year-old Afghan man, migrated to Canada with his daughter’s family after fleeing political unrest. Though he brought some financial resources, preserving a degree of independence, he struggles with the loss of his former identity as a respected teacher. Now reliant on his daughter’s household, he feels a loss of agency, despite managing his own finances. Taher stays connected to his culture through community gatherings, religious events, and frequent contact with extended family. He faces age-related health issues but proactively seeks care with support. While intergenerational gaps sometimes leave him feeling unheard, his cultural and religious engagement provides emotional grounding. Taher lives between two worlds and being nostalgic for the past yet partially integrated into his present life.
Contrary case: A case that is completely absent of the concept’s defining features [30].	Zahra, a 79-year-old Sudanese immigrant in Australia, has fully adapted to her new environment, maintaining a strong sense of identity, agency, and belonging. She embraces her cultural roots through active participation in her community and regular visits to Sudan, viewing migration as an opportunity rather than a loss. Zahra plays a respected role within her family, helping with childcare and household decisions. With stable health, economic independence, and confidence in navigating healthcare, she remains a valued and active member of both her family and community.

## Data Availability

The original contributions presented in this study are included in the article/Supplementary Material.

## Supplementary Materials

The following supporting information can be downloaded at: https://www.mdpi.com/article/10.3390/healthcare13202606/s1, Data Extraction Table [116,117,118,119,120,121,122,123,124,125,126,127,128,129,130].

## Appendix A. Refined Search Strategy with the Medical Subject Headings (MeSH) Terms

**Ambiguous Loss Concepts**	**Older Adult Terms**	**Migration Terms**
Loss	“Older adult”	“Migrant”
“Ambiguous loss”	“Aging”	“Refugee”
“Unresolved loss”	“Elderly”	“Displaced person”
“Non-death loss”	“Senior”	“Asylum seeker”
“Migration and loss”	“Older person”	“Immigrant”
“Psychosocial loss”	“Geriatrics”	“Undocumented migrant”
“Physical loss”	“Late-life”	
“Family separation”		
“Loss and identity”		

## Appendix B. Database Search

**Database**	**Interface**	**Search String**	**Limiters**	**Findings**
Medline	Ovid	(“Loss” or “Ambiguous loss” or “Unresolved loss” or “Non-death loss” or “Migration and loss” or “Psychosocial loss” or “Physical loss” or “Family separation” or “Loss and identity”) AND (Migrant* or Refugee* or “Displaced person*” or “Asylum seeker*” or Immigrant* or “Undocumented migrant*”) AND (“Older adult$” or Aging or Elderly or Senior$ or “Older person$” or Geriatrics or “Late-life”).mp. [mp = title, book title, abstract, original title, name of substance word, subject heading word, floating sub-heading word, keyword heading word, organism supplementary concept word, protocol supplementary concept word, rare disease supplementary concept word, unique identifier, synonyms, population supplementary concept word, anatomy supplementary concept word]	limit 4 to (English language and yr = “2010”)	5
CINAHL	EBASCOhost	(“loss” OR “Ambiguous loss” OR “Unresolved loss” OR “Non-death loss” OR “Migration and loss” OR “Psychosocial loss” “Physical loss” OR “Family separation” OR “Loss and identity”) AND (Migrant? OR Refugee? OR “Displaced person?” OR “Asylum seeker?” OR Immigrant? OR “Undocumented migrant?”) AND (“Older adult$” OR Aging OR Elderly OR Senior$ OR “Older person$” OR Geriatrics OR “Late-life”)	Publication Date: 20100101-; English Language	43
PsycINFO	EBASCOhost	(“loss” OR “Ambiguous loss” OR “Unresolved loss” OR “Non-death loss” OR “Migration and loss” OR “Psychosocial loss” “Physical loss” OR “Family separation” OR “Loss and identity”) AND (Migrant? OR Refugee? OR “Displaced person?” OR “Asylum seeker?” OR Immigrant? OR “Undocumented migrant?”) AND (“Older adult$” OR Aging OR Elderly OR Senior$ OR “Older person$” OR Geriatrics OR “Late-life”)	Publication Date: 20100101-; English Language	82
Scopus	Scopus (Elsevier)	(TITLE-ABS-KEY(“Loss” OR “Ambiguous loss” OR “Unresolved loss” OR “Non-death loss” OR “Migration and loss” OR “Psychosocial loss” OR “Physical loss” OR “Family separation” OR “Loss and identity”)) AND (TITLE-ABS-KEY(Migrant* OR Refugee* OR “Displaced person*” OR “Asylum seeker*” OR Immigrant* OR “Undocumented migrant*”)) AND (TITLE-ABS-KEY(“Older adult*” OR Aging OR Elderly OR Senior* OR “Older person*” OR Geriatrics OR “Late-life”))	Publication Date: 20100101-; English Language	237
