# Supplementary material for: Ambiguous Loss Among Aging Migrants: A Concept Analysis- and Nursing Care-Oriented Model
Source: Healthcare (Basel). 2025 Oct 16;13(20):2606. doi: 10.3390/healthcare13202606 (PMC12562954; doi:10.3390/healthcare13202606)
Supplement: Supplementary file 1 [file healthcare-13-02606-s001.zip › healthcare-3890028-supplementary.pdf]

| Article Name | Authors | Year of Publication | Relevant Concepts (i.e., keywords, key concepts discussed) | Antecedents: Events or situations that need to happen before the concept can occur.<br>What is required for the concept to occur? (Factors that affect, stimulate or encourage the concept to occur) | Defining Attributes: Precise terms that can be used to clarify a concept, aiding in differentiating it from other similar concepts | Consequences: The events or situations that may arise following the manifestation of a concept, frequently inspiring fresh perspectives or research paths related to a specific concept. (The outcomes as a result of the concept) | Empirical Referents: Are measurable occurrences of the phenomenon that facilitate the concept's quantification. (i.e. the name of theory, framework, scale for measurement, survey) | Citation (APA 7th Edition) | Notes |
|---|---|---|---|---|---|---|---|---|---|
| **Unveiling Social Belonging: Exploring the Narratives of Immigrant Muslim Older Women** | Au, A.; Hilario, C.; Meherali, S.; Salma, J. | 2024 | | - Migration<br>- Islamaphobia, religious and racial discrimination<br>- Aging | - Loss of sense of belonging<br>- Leaving behind loved ones<br>- Aging/growing old<br>- Loss of social networks<br>- Death of family | - Avoidant behaviours: avoiding feeling lonely or isolated by engaging with others<br>- Protective coping behaviours such as social engagement (i.e., friends, family, neighbours, peers), increased motivation to strengthen relationships with others<br>- Identifying "chosen families", through community, same-language peers, checking in on others<br>- Increased loneliness, isolation<br>- Fear of dying<br>- Increased social gatherings, peer-to-peer support<br>- Increased community engagement, leading to sense of purpose and contribution<br>- Adjusting lifestyle to improve family dynamics, cultivating positivty, self-awareness, emotional regulation<br>- Increased family support<br>- Increased intergenerational engagement<br>- Sense of gratitude | - Integrative Framework for Belonging<br>- descriptive, narratives, photo elicitation interviews | Au, A., Hilario, C., Meherali, S., Salma, J., & Tracy Collins. (2024). Unveiling Social Belonging: Exploring the Narratives of Immigrant Muslim Older Women. Health & Social Care in the Community, 2024(1). https://doi.org/10.1155/2024/5598247 | |
| **“We Used to Have Four Seasons, but Now There Is Only One”: Perceptions Concerning the Changing Climate and Environment in a Diverse Sample of Israeli Older Persons** | Ayalon, L.; Ulitsa, N.; AboJabel, H.; Engdau-Vanda, S. | 2024 | solastalgia, nostalgia, environmental changes, climate change, older persons | - Climate change<br>- Pollution<br>- Immigration | - Loss of physical and natural environments | - Increased sense of loss and longing for what was lost (solastalgia and nostalgia)<br>- Grief<br>- Emotional distress | - Interviews and focus groups | Ayalon, L., Ulitsa, N., AboJabel, H., & Engdau-Vanda, S. (2024). “We Used to Have Four Seasons, but Now There Is Only One”: Perceptions Concerning the Changing Climate and Environment in a Diverse Sample of Israeli Older Persons. Journal of Applied Gerontology, 43(5), 527–535. https://doi.org/10.1177/07334648231212279 | |
| **Digital mourning on Facebook: the case of Filipino migrant worker live-in caregivers in Israel** | Babis, D. | 2021 | digital mourning, Filipinos, Israel, live-in caregivers for the elderly, migrant workers,<br>online grief | | - Loss of employer;<br>- Loss of migrant workers | - "Digital mourning/grief": mourning on social media networks, expressing and sharing grief online via posts; posting memories, condolescenes online<br>- "Communial digital mourning"<br>- Sense of belonging<br>- Community building | - Digital ethnography, data capture of Facebook posts | Babis, D. (2021). Digital mourning on Facebook: the case of Filipino migrant worker live-in caregivers in Israel. Media, Culture & Society, 43(3), 397–410. https://doi.org/10.1177/0163443720957550 | |
| **Transnational Ageing, Intergenerational Family Ties and the Social Embedding of Older Italian Migrants in Australia** | Brandhorst, R. | 2023 | | - Admission into residential care home, separation from family and friends<br>- Memory loss<br>- Death of loved ones<br>- Diminishing health<br>- Transnational aging | - Loss/death of significant other<br>- Decreased social embedding<br>- Decreased English language knowledge<br>- Decreased self-trust<br>- Decreased physical (auditory and visual) and cognitive capacities, physical immobility | - Decreased social embedding, decreased belonging, increased nostalgia, increased reliance on family<br>- Enhanced trust and reliance on children as support<br>- Increased reliance on face-to-face contact and physical presence for care | Case study, participant observation, interviews | Brandhorst, R., Cienfuegos, J., & Fahy Bryceson, D. (2023). Transnational Ageing, Intergenerational Family Ties and the Social Embedding of Older Italian Migrants in Australia. In Handbook of Transnational Families Around the World (pp. 187–202). Springer International Publishing. https://doi.org/10.1007/978-3-031-15278-8_12 | |
| **Older Vietnamese refugees’ transnational digital social capital and its impact on social inclusion** | Brandhorst, R. | 2024 | Digital capital; social capital;<br>refugee studies;<br>transnational care;<br>transnational ageing | - Forced displacement<br>- Transnational immigration<br>- War<br>- Risk for political persecution, ethnic persecution; violence<br>- Escaping home country<br>- Exile | - Loss of economic and material capital<br>- Loss of personal properties<br>- Loss of social capital | - Greater reliance on social capital<br>- "transnational digital social capital"<br>- Increased digital literacy<br>- Construction of diasporic identity<br>- Reliance on social media to re-connect with distance relatives/social network<br>- Increase use of information and communicaton technology | Bourdieu’s capital approach, narrative-biographical, ethnographic and qualitative network<br>analytical study; case studies | Brandhorst, R. (2024). Older Vietnamese refugees’ transnational digital social capital and its impact on social inclusion. Journal of Ethnic and Migration Studies, 50(18), 4642–4660. https://doi.org/10.1080/1369183X.2023.2208738 | |
| **The Impact of the Covid-19 Pandemic and the Lockdown on the Health and Living Conditions of Undocumented Migrants and Migrants Undergoing Legal Status Regularization** | Burton-Jeangros, C.; Duvoisin, A.; Lachat, S.; Consoli, L.; Fakhoury, J.; Jackson, Y. | 2020 | COVID-19, migrant, impact, lockdown, undocumented, health, living conditions | - pandemic and lockdown<br>- informal and precarious employment<br>- food insecurity<br>- immigration status (undocumented)<br>- unwillingness to seek help from government or official sources | - Loss of employment and income<br>- Hunger | - increased negative health outcomes due to COVID, anxiety, depression<br>- avoidance/decrease health-seeking behaviours<br>- financial, housing, food insecurity<br>- decreased life satisfaction, self-rated health<br>- seeking financial support from friends and peers through loans<br>- changing in coping strategies<br>- decreased psychological health<br>- reluctance to seek support from formal services or government | cross-sectional mixed methods study; self-rated health ( 12-Item Short-Form Survey (SF-12)); | Burton-Jeangros, C., Duvoisin, A., Lachat, S., Consoli, L., Fakhoury, J., & Jackson, Y. (2020). The Impact of the Covid-19 Pandemic and the Lockdown on the Health and Living Conditions of Undocumented Migrants and Migrants Undergoing Legal Status Regularization. Frontiers in Public Health, 8, 596887–596887. https://doi.org/10.3389/fpubh.2020.596887 | |

| Article Name | Authors | Year of Publication | Relevant Concepts (i.e., keywords, key concepts discussed) | Antecedents: Events or situations that need to happen before the concept can occur. What is required for the concept to occur? (Factors that affect, stimulate or encourage the concept to occur) | Defining Attributes: Precise terms that can be used to clarify a concept, aiding in differentiating it from other similar concepts | Consequences: The events or situations that may arise following the manifestation of a concept, frequently inspiring fresh perspectives or research paths related to a specific concept. (The outcomes as a result of the concept) | Empirical Referents: Are measurable occurrences of the phenomenon that facilitate the concept's quantification. (i.e. the name of theory, framework, scale for measurement, survey) | Citation (APA 7th Edition) | Notes |
|---|---|---|---|---|---|---|---|---|---|
| **The Impact of the COVID-19 Pandemic on Older Latino Immigrants** | Calvo, R.; Waters, M. C. | 2023 | older adults, COVID-19, Latino, late- age, immigrants | - pandemic and lockdown<br>- immigration status, ineligibility for formal services or assistance programs; lack of access to benefits, financial aid, COVID relief funds, employment insurance<br>- "Legacy of cumulative disadvantage": Lack of/existing financial security prior to the pandemic, precarious work, poorly paid, ineligibility of social security pension<br>- Recency of immigration (immigrants who have lived longer, established legal status were more likely to be well off)<br>- Legilation that target migrants: risk of "public charge", disqualification from benefits, pensions, legalization | - Loss of employment, income<br>- Hunger, loss of food<br>- Exclusion from federal aid<br>- Loss of working hours<br>- Lack of access to healthcare | - Increased use of food pantries<br>- Financial and food insecurity<br>- Reliance on non-profits, community organizations<br>- Increased social isolation<br>- Increased reliance on children and family for support<br>- Financial redistribution (sharing costs and pension), sacrificing food and living conditions for betterment of family members<br>- Increased reliance on home remedies<br>- Increased pooling of resources | semi-structured interviews | CALVO, R., & WATERS, M. C. (2023). The Impact of the COVID-19 Pandemic on Older Latino Immigrants. RSF : Russell Sage Foundation Journal of the Social Sciences, 9(3), 60–76. https://doi.org/10.7758/RSF.2023.9.3.03 | |
| **What is frailty? Perspectives from Chinese clinicians and older immigrants in New Zealand': Correction** | Gary Cheung1 & Susan Gee 2 & Hamish Jamieson2 & Ulrich Berger3 | 2021 | Ethnicity . Frailty . Health professionals. Older people . Overseas Chinese | - Aging, older age | - Loss of confidence<br>- Frailty ("loss of ... energy, physical ability, cognition, and health")<br>- Experience of aging as a loss itself ("Loss of health...friends...family members") | - decreased health outcomes, increased polypharmacy, increased medical comorbidities and chronic illness<br>- increased weakness, decline in functioning (physical, cognitive), increased risk for cognitive impairment, hinders activities of daily living, increased exhaustion, decreased activity<br>- Effects on psychological and social health: depression, mental health, decreased perceived control, | focus groups;<br>Chinese-Canadian study of health and aging clinical frailty scale physician version (CSHA-CFS PV)<br><br>FRAIL scale<br><br>"Malmstrom and Morley (2013) developed a scale "SOCIAL" to screen for psychosocial risk factors for frailty: Sadness, Outside activity, Cognition, Income adequacy, Attachment to neighbour, Lethargy" | Cheung, G., Gee, S., Jamieson, H. A., & Bergler, H. U. (2021). Correction to: What Is Frailty? Perspectives from Chinese Clinicians and Older Immigrants in New Zealand. Journal of Cross-Cultural Gerontology, 36(2), 215–215. https://doi.org/10.1007/s10823-021-09429-9 | |
| **Post-war immigration experiences of survivors of the Korean war** | Cho Kim, Sara; Manchester, Chelsea; Lewis, Ariel | 2019 | Immigration; Asian American; war trauma; aging population; intergenerational | - War, post-war<br>- Political corruption; changing political regimes<br>- Leaving homeland to seek better life abroad<br>- Separation of families<br>- Exposure to foreign cultures, acculturation, conformity<br>- Uncertainty, navigating foreign culture vs own culture and which to adopt<br>- Shifting in personal perspectives and thinking<br>- Children adopting Western lifestyle/acculturation<br>- Wish to start a new life<br>- Displacement | - Loss of identity (loss of family traditions)<br>- Loss of male authoritive figures, husband and fathers, family members<br>- Loss of social class/standing<br>- Loss of privacy and space<br>- Loss of land | - Generational trauma<br>- Changing family dynamics, changing parenting styles | interview | Cho Kim, S., Manchester, C., & Lewis, A. (2019). Post-War Immigration Experiences of Survivors of the Korean War. Journal of Aggression, Maltreatment & Trauma, 28(8), 977–995. https://doi.org/10.1080/10926771.2017.1392388 | |
| **Understanding hearing loss and barriers to hearing health care among Korean American older adults: A focus group study** | Choi, Janet S.; Shim, Kyoo S.; Kim, Kunhwa; Nieman, Carrie L.; Mamo, Sara K.; Lin, Frank R.; Han, Hae-Ra | 2018 | hearing loss, Korean American, hearing health care, hearing aids, immigrant health | Aging process | - Hearing | - Increased frustration, difficulties in communication, stress, discomfort, personal shame<br>- Decreased self-confidence<br>- Impaired understanding<br>- Changing coping strategies: increased technology use, pretending to hear, social withdrawal/avoiding social situations; | - focus groups | Choi, J. S., Shim, K. S., Kim, K., Nieman, C. L., Mamo, S. K., Lin, F. R., & Han, H.-R. (2018). Understanding Hearing Loss and Barriers to Hearing Health Care Among Korean American Older Adults: A Focus Group Study. Journal of Applied Gerontology, 37(11), 1344–1367. https://doi.org/10.1177/0733464816663554 | |
| **Later life migration: Sociocultural adaptation and changes in quality of life at settlement among recent older Chinese immigrants in Canada** | Da, Wei-Wei; Garcia, Alicia | 2015 | Socio-cultural adaptation, quality of life, older Chinese immigrants, Canada | - Co-habitation with children, lack of independent home<br>- Migration | - Loss of decision-making power, independence<br>- Loss of home ownership<br>- Loss of social support networks<br>- Loss of opportunities for socialization<br>- Loss of resources<br>- Loss of connection with those left behind<br>- Loss of familiar setting | - Increase socialization through involvement in religious activities and attending English language classes | - structured interviews | Da, W.-W., & Garcia, A. (2015). Later Life Migration: Sociocultural Adaptation and Changes in Quality of Life at Settlement Among Recent Older Chinese Immigrants in Canada. Activities, Adaptation, & Aging, 39(3), 214–242. https://doi.org/10.1080/01924788.2015.1063330 | |
| **Aging Filipina migrants' experiences of transnational end-of-life care and loss over time** | de Leon, C.; Blower-Nassiri, J. | 2024 | Filipino migrants; transnational families; end-of-life care; aging; loss; COVID-19 | - Living away from homeland; experiences of loss from a distance | - Loss of loved ones (from a distance)<br>- Accumulated losses<br>- Symbolic losses, loss of language, location, family<br>- "accrual of loss over time" | - reliance on technology for communication and digital means of care<br>- changing roles of transnational kin, children as proxys and mediators of grieving practices<br>- reciprocation of care over long distances | "kuwentuhan" story telling; journal entries; | de Leon, C., & Blower-Nassiri, J. (2024). Aging Filipina migrants' experiences of transnational end-of-life care and loss over time. Ethnic and Racial Studies, 47 (14), 3064–3083. https://doi.org/10.1080/01419870.2024.2351636 | |

| Article Name | Authors | Year of Publication | Relevant Concepts (i.e., keywords, key concepts discussed) | Antecedents: Events or situations that need to happen before the concept can occur.<br>What is required for the concept to occur? (Factors that affect, stimulate or encourage the concept to occur) | Defining Attributes: Precise terms that can be used to clarify a concept, aiding in differentiating it from other similar concepts | Consequences: The events or situations that may arise following the manifestation of a concept, frequently inspiring fresh perspectives or research paths related to a specific concept. (The outcomes as a result of the concept) | Empirical Referents: Are measurable occurrences of the phenomenon that facilitate the concept's quantification. (i.e. the name of theory, framework, scale for measurement, survey) | Citation (APA 7th Edition) | Notes |
|---|---|---|---|---|---|---|---|---|---|
| **I feel like her daughter not her mother': Ethnographic trans-cultural perspective of the experiences of aging for a group of Southeast Asian refugees in the United States** | Dubus, Nicole | 2010 | Cambodian<br>Group work<br>Refugees<br>Trans-cultural aging | - Refugees, war, fear of persecution, forceful migration<br>- Acculturation of children to Western perspectives, leading to lack of respect, intergenerational differences, children treating parents poorly<br><br>- PTSD exacerbation years after event | - Loss of social roles (respected elder)<br>- Loss of relationship with children, loss of respect<br><br>- Loss of employment<br><br>- Fear of living alone as a woman | - Anxiety, depression, feelings of fear, uncertainty, worry, frustration, sadness<br>- Feeling devalued<br>- Grieving loss of social role<br>- Feeling out of place<br>- Feeling unprepared for aging (experiences not aligning with personal expectations and perceptions of aging)<br>- Fear of living alone without support from children | ethnography | Dubus, N. (2010). "I feel like her daughter not her mother": Ethnographic trans-cultural perspective of the experiences of aging for a group of Southeast Asian refugees in the United States. Journal of Aging Studies, 24(3), 204–211. https://doi.org/10.1016/j.jaging.2010.02.002 | |
| **Forced migration-induced diminished social networks and support, and its impact on the emotional wellbeing of older refugees in Western countries: A scoping review** | Ekoh, Prince Chiagozie; Iwuagwu, Anthony Obinna; George, Elizabeth Onyedikachi; Walsh, Christine A. | 2023 | Social networks<br>Social support<br>Older refugees<br>Wellbeing<br>Migration-induced loss | - Migration, forced migration<br>- Separation from friends and family | - Loss of social networks and support from friends and family | - Risk of unaddressed care needs<br>- Difficulty in building resilience<br>- Loneliness, isolation<br>- Difficulty accessing care, gaps in service access<br>- Sadness<br>- Challenges to resettlement and integration<br>- Increased stress<br>- Mental health issues: increased depression, grief, increased PTSD symptoms and severity, anxiety<br>- Decreased well-being, quality of life, life satisfaction | Scoping review | Ekoh, P. C., Iwuagwu, A. O., George, E. O., & Walsh, C. A. (2023). Forced migration-induced diminished social networks and support, and its impact on the emotional wellbeing of older refugees in Western countries: A scoping review. Archives of Gerontology and Geriatrics, 105, 104839–104839. https://doi.org/10.1016/j.archger.2022.104839 | |
| **Perceptions of health-related quality of life (HRQOL) experienced by older ethnic Somalis aging transculturally in the US: An interpretative phenomenological analysis** | Evans, Shelly D. | 2022 | | - Migration<br>- Seperation from family<br>- Conflict | - Loss of social status<br>- Loss of dignity<br>- Loss of family members, death of family<br>- Loss of homeland, shared culture, common language, assets<br>- Loss of resources | - Grief<br>- Survivor's guilt<br>- Decreased health-related quality of life<br>- Decreased self-worth | Interpretive phenomenology; WHO Quality of Life | Evans, S. D. (2021). Perceptions of Health-Related Quality of Life (HRQoL) Experienced by Older Ethnic Somalis Aging Transculturally in the U.S.: An Interpretative Phenomenological Analysis. ProQuest Dissertations & Theses. | |
| **Suicide Risk among Immigrants and Ethnic Minorities: A Literature Overview** | Forte, A.; Trobia, F.; Gualtieri, F.; Lamis, D. A.; Cardamone, G.; Giallonardo, V.; Fiorillo, A.; Girardi, P.; Pompili, M. | 2018 | immigrants; ethnic minorities; suicide; prevention | - Language barrier<br>- Family separation | - Loss of status, social network<br>- Lack of information on healthcare system | - Increased suicide risk, suicidal behaviour, death | - Literature review | Forte, A., Trobia, F., Gualtieri, F., Lamis, D. A., Cardamone, G., Giallonardo, V., Fiorillo, A., Girardi, P., & Pompili, M. (2018). Suicide Risk among Immigrants and Ethnic Minorities: A Literature Overview. International Journal of Environmental Research and Public Health, 15(7), 1438-. https://doi.org/10.3390/ijerph15071438 | |
| **Forty years in Aotearoa New Zealand: White identity, home and later life in an adopted country** | George, Molly; Fitzgerald, Ruth P. | 2012 | migration, globalisation, transnationalism, ageing in place,<br>home. | - Unaffordable travel, frailty hindering travel, lack of financial resources<br>- Passage of time | - Loss of immigrant friends<br>- Loss of ability to return home<br>- Loss of home, identity | - Feeling of separation | Interviews, narrative inquiry | GEORGE, M., & FITZGERALD, R. P. (2012). Forty years in Aotearoa New Zealand: white identity, home and later life in an adopted country. Ageing and Society, 32(2), 239–260. https://doi.org/10.1017/S0144686X11000249 | |
| **The immigration experience among elderly Egyptian immigrants in the United States** | Girgis, Ihab | 2016 | | - Migration, risk for persecution, seeking asylum<br>- Separation from familiar environment<br>- Aging | - Loss of:<br>- familiar environment<br>- relationships<br>- social status and roles<br>- independence<br>- control<br>- customs and traditions | - Emotional distress<br>- Grief<br>- Homesickness, missing family<br>- "create a void"<br>- Feelings of regret about migration | Interviews, descriptive phenomenology, reflexive methodology, collective case study<br>Immigration theories: segmented assimilation theory<br>Aging theories: disengagement, continuity, Erikson's stages of psychosocial development, social intergration, life course<br>Stress and coping theories: | Girgis, I. (2015). The immigration experience among elderly Egyptian immigrants in the United States. ProQuest Dissertations & Theses. | |
| **COVID-19 lockdown and penalty of joblessness on income and remittances: A study of inter-state migrant labourers from Assam, India** | Guha, P.; Islam, B.; Hussain, M. A. | 2021 | | - pandemic and lockdown | - Loss of income and employment | - Increased financial hardship | Telephone survey; | Guha, P., Islam, B., & Hussain, M. A. (2021). COVID-19 lockdown and penalty of joblessness on income and remittances: A study of inter-state migrant labourers from Assam, India. Journal of Public Affairs, 21(4), e2470-n/a. https://doi.org/10.1002/pa.2470 | |
| **Unveiling the War Child Syndrome: Finnish War Children's Experiences of the Evacuation to Sweden During WWII from a Lifetime Perspective** | Heilala, Cecilia; Santavirta, Nina | 2016 | Early separation;<br>evacuation;<br>shame; trauma | - early childhood separation from families, evacuation, foster care<br>- war child syndrome<br>- Lack of information, uncertainty with evacuation<br>- Separation from home, then subsequent separation from foster care | - Loss of confidence<br>- Loss of self-worth<br>- "Rootlessness, detachment"; lack of strong tie or connection a place or community | - Feelings of regret<br>- Difficulty in adjusting into new lifestyles and with new family (e.g., foster parents)<br>- Fear of rejection, feelings of unworthiness<br>- Fear, loneliness, worry<br>- Feelings of abandonmen, guilt, and shame | Short Form-36 scale measuring health and well-being; Survey | Heilala, C., & Santavirta, N. (2016). Unveiling the War Child Syndrome: Finnish War Children's Experiences of the Evacuation to Sweden During WWII from a Lifetime Perspective. Journal of Loss & Trauma, 21(6), 575–588. https://doi.org/10.1080/15325024.2016.1161425 | |

| Article Name | Authors | Year of Publication | **Relevant Concepts** (i.e., keywords, key concepts discussed) | **Antecedents:** Events or situations that need to happen before the concept can occur.<br>What is required for the concept to occur? (Factors that affect, stimulate or encourage the concept to occur) | **Defining Attributes:** Precise terms that can be used to clarify a concept, aiding in differentiating it from other similar concepts | **Consequences:** The events or situations that may arise following the manifestation of a concept, frequently inspiring fresh perspectives or research paths related to a specific concept. (The outcomes as a result of the concept) | **Empirical Referents:** Are measurable occurrences of the phenomenon that facilitate the concept's quantification. (i.e. the name of theory, framework, scale for measurement, survey) | **Citation (APA 7th Edition)** | **Notes** |
|---|---|---|---|---|---|---|---|---|---|
| **Transnational Caregiving and Grief: An Autobiographical Case Study of Loss and Love During the COVID-19 Pandemic** | Hinkson, G. M.; Huggins, C. L.; Doyle, M. | 2024 | COVID-19, transnational caregiving, complicated grief, disenfranchised grief, older<br>adults | - pandemic and lockdown, physical separation, routine disturbances<br>- unexpected death<br>- transnational caregiving | - Loss of traditions and rituals related to deceased loved ones<br>- Loss of ability to be present during loved ones death<br>- "Disenfranchised grief"<br>- Ambiguous loss (Boss 2022)<br>- Loss of physical presence of family members during experiences of dying | - Adoption of caregiving role<br>- Isolation, helplessness<br>- Grief and mourning<br>- Anxiety, regret, sense of failure, loss of control, survivor's guilt<br>- Lack of closure<br>- "misbereavement"<br>- Transnational caregiving | Qualitative case studies, autobiographical case studies ; semistructured interviews | Hinkson, G. M., Huggins, C. L., & Doyle, M. (2024). Transnational Caregiving and Grief: An Autobiographical Case Study of Loss and Love During the COVID-19 Pandemic. Omega: Journal of Death and Dying, 90(1), 5–20. https://doi.org/10. 1177/00302228221095689 | |
| **Spiral Loss of Culture: Cultural Trauma and Bereavement of Bhutanese Refugee Elders** | Im, H.; Neff, J. | 2021 | acculturation; Bhutanese refugees; cultural bereavement; cultural trauma; loss spiral; qualitative research; refugee<br>elders; resettlement; stress & coping | - Migration and resettlement, forced migration<br>- Sudden change in environment, culture shock, shifts in lifestyle, Westernized perspectives<br>- lack of technological skills, adapting to urban lifestyles<br>- adjusting to host country cultural norms and lifestyles<br>- Language barriers<br>- Pressure to assimilate | - Loss of culture (farming, traditions, agriculture, community hospitality), loss of self-identity and share identitiy<br>- Loss of independence, coping mechanisms, resources<br>- Barriers to healthcare access, unaddressed health needs<br>- Loss of language<br>- Loss of cultural practices in younger generations | - Intergenerational gaps, acculturative gaps<br>- Grief<br>- Trauma<br>- Uncertainty around transmission of religion, traditions and cultures across future generations<br>- Decreased support from children<br>- Difficulty in mantaining a sense of belonging<br>- Stressors with acculturation<br>- distress<br>- Lack of coping mechanisms and capacity to cope,<br>- burdens on families, family tensions, conflict | - focus group interviews | Im, H., & Neff, J. (2021). Spiral Loss of Culture: Cultural Trauma and Bereavement of Bhutanese Refugee Elders. Journal of Immigrant & Refugee Studies, 19(2), 99–113. https://doi. org/10.1080/15562948.2020.1736362 | |
| **Aging, memory loss, and Alzheimer's disease: What do refugees from the former Soviet Union think?** | Iris, Madelyn; Schrauf, Robert W. | 2017 | Aging/ageing; Alzheimer's disease; memory loss; Russian-speaking refugees | - Aging, older age<br>- Unfamiliar environment<br>- Stress from trauma, war, hunger, tragedy | - Memory loss, Alzheimer's<br>- Loss of occupation, social status, professional status, independence, | - Depression, anxiety, stress<br>- Isolation, dependency | - Interviews | Iris, M., & Schrauf, R. W. (2017). Aging, memory loss, and Alzheimer's disease: What do refugees from the former Soviet Union think? Journal of Religion, Spirituality & Aging, 29(2–3), 130–146. https://doi.org/10.1080/15528030. 2016.1169568 | |
| **Social isolation and loneliness among immigrant and refugee seniors in Canada: a scoping review** | Johnson, Shanthi; Bacsu, Juanita; McIntosh, Tom; Jeffery, Bonnie; Novik, Nuelle | 2019 | Canada, Social isolation, immigrant | - Migration<br>- Social isolation and loneliness<br>- Bereavement, death of loved ones<br>- Changes in cultural norms and practices | - Sense of loss<br>- Loss of autonomy<br>- Loss of social networks, language<br>- Loss of familial values and filial piety | - Nostalgia and homesickness, longing for homeland<br>- Coping strategies: increased social interaction, engaging in social activities like volunteering, visiting homeland<br>- Social isolation, loneliness<br>- Limited information, access to services, | - Literature search, scoping review | Johnson, S., Bacsu, J., McIntosh, T., Jeffery, B., & Novik, N. (2019). Social isolation and loneliness among immigrant and refugee seniors in Canada: a scoping review. International Journal of Migration, Health and Social Care, 15(3), 177–190. https://doi.org/10. 1108/IJMHSC-10-2018-0067 | |
| **Barriers to and facilitators of diabetes self-management with elderly Korean-American immigrants** | Joo, J. Y.; Lee, H. | 2016 | barriers, facilitators, elderly, immigrants, type 2 diabetes, language, self-control, limited resources, patient centered nursing, disease management | diagnosis of type 2 DM, cultural background, limited english proficiency, healthcare systen challenges, inadequate social/family support, health beliefs of diabetes, financial instability, psychologcial well being, physical functionality | the high cost of medications/care, language issues, loss of self, memory loss, and limited healthcare access, poor hea;th literacy, cultural diet, time information, support | patient-centred, age and culturally appropriate diabetes interventions, social supports, tanslatopрs, enhanced/decreased QOL, improved/poor T2DM managment, reduced hospitalization, cultural adaptation, timely/delayed care. dietary/lifestyle alterations | focus groups, individual interviews, questionnaire, transcription, transaltion, korean, english | Joo, J. Y., & Lee, H. (2016). Barriers to and facilitators of diabetes self-management with elderly Korean-American immigrants. International Nursing Review, 63(2), 277–284. https://doi.org/10. 1111/inr.12260 | |
| **Transnational daughters in Australia: Caring remotely for ageing parents during COVID 19** | Joseph, Dawn; Belford, Nish; Lahiri-Roy, Reshmi | 2022 | Aging, parents, migration, emotional reflexivity, remote caring, daughters, contact, culture, collaborative autoethnography | Migration and Transnational Livin, Aging parents in home countries, COVID-19 restrctions, cultural normas and family values | **Emotional reflexivity** , digitaal communication, collaborative autoethnography, integration of creative/cultural disciplaines | psychologcial stress and emotional strain, strengthened familial bonds, development of resilient coping mechanisms, contribition and valuable insights, improved caregiving dynamics, loss, fear grief, | dialogue, conversation, autoethnographical recounts, emotionally reflective theoretical lens | Joseph, D., Belford, N., & Lahiri-Roy, R. (2022). Transnational daughters in Australia: Caring remotely for ageing parents during COVID 19. Emotion, Space and Society, 42, 100864-. https://doi. org/10.1016/j.emospa.2021.100864 | |
| **Displaced Selves: Older African Adults in Forced Migration** | Kilpeläinen, F.; Zechner, M. | 2022 | older adults, African Refugees, diapolcemnt, migration | foeced migration events, cultural/social toles, age rekated challenges, health adaptation, language and communication barriers | identity continuity/discontinuity, coping strategies, self perceptions, social group/community engagement | psychological well being, social integration, resilience development, cultural preservation. | phenomenology, abductive thematic analysis, semi-structured interviews, identity process theories | Kilpeläinen, F., & Zechner, M. (2022). Displaced Selves: Older African Adults in Forced Migration. *Journal of Refugee Studies*, 35(3), 1126–1142. https://doi. org/10.1093/jrs/feac046 | |
| **Content and Intensity of Pride and Regret Among Asian American Immigrant Elders** | Lee, Othelia E.; Ryu, Seungah | 2017 | emotions, immigrants, regret, pride, adults, life events, self-conscious | immigraton experience. deep rooted cultural values, life course events, achievemtns, failures, role shifts | Pride- educational/career achievements, family and social contrubutions<br>Regret- unfuldilled aspirations, nsuccessful goals, family dynamic regrets | **Emotional wellbeing, intergenerational relationbships, cultural idenitty reinfiircment** | community recruitment, cross-sectional, semistructured, face-to-face interviews, convenience sample, translation, mixed-methods approach | Lee, O. E., & Ryu, S. (2017). Content and Intensity of Pride and Regret Among Asian American Immigrant Elders. *Illness, Crisis, and Loss*, 25(4), 309–322. https://doi. org/10.1177/1054137317723103 | |
| **What do Korean American immigrants know about Alzheimer's disease (AD)? The impact of acculturation and exposure to the disease on AD knowledge** | Lee, Sang E.; Lee, Hee Yun; Diwan, Sadhna | 2010 | literacy, language, chronic illness, culture, education, steretypes, myths | acculturation level, prior exposure to alzheimers, educational background, langiage proficiency | knowledge of alzheimers, cultural perveptions of dementia, healthcare utilization patterns, healthcare acculturation | early detection/intervention, caregiving preparedness, reduction in stigma, improved quality of life | self-administered questionnaire, a quota sampling method | Lee, S. E., Lee, H. Y., & Diwan, S. (2010). What do Korean American immigrants know about Alzheimer's disease (AD)? The impact of acculturation and exposure to the disease on AD knowledge. *International Journal of Geriatric Psychiatry,* 25(1), 66–73. https://doi. org/10.1002/gps.2299 | |
| **Art in health and identity: Visual narratives of older Chinese immigrants to New Zealand** | Li, Wendy Wen | 2012 | identity, health, art, aesthetics, value, immigrants, Chinese, loss, anmxiety, culture, | immiogration and cultural transition, loss of familar social roles, desire for expression and connection, biographical disruption and status-discrepancy | identity reconstruction, visual narratives, art making as a coping mechanism, cultrual transitions, healyhy adaptation | **enhanced well being, cultural/biographical continuity, social integration, reovcery, restoration, semnse pf control** | semi-structured interviews, visual narratives, photography, thematic analyssi, isual analysis proposed by Riessman (Citation2008) | Li, W. W. (2012). Art in health and identity: Visual narratives of older Chinese immigrants to New Zealand. *Arts & Health*, 4(2), 109–123. https://doi. org/10.1080/17533015.2011.584886 | |

| Article Name | Authors | Year of Publication | **Relevant Concepts** (i.e., keywords, key concepts discussed) | **Antecedents:** Events or situations that need to happen before the concept can occur.<br>What is required for the concept to occur? (Factors that affect, stimulate or encourage the concept to occur) | **Defining Attributes:** Precise terms that can be used to clarify a concept, aiding in differentiating it from other similar concepts | **Consequences:** The events or situations that may arise following the manifestation of a concept, frequently inspiring fresh perspectives or research paths related to a specific concept. (The outcomes as a result of the concept) | **Empirical Referents:** Are measurable occurrences of the phenomenon that facilitate the concept's quantification. (i.e. the name of theory, framework, scale for measurement, survey) | Citation (APA 7th Edition) | Notes |
|---|---|---|---|---|---|---|---|---|---|
| **Loss of friends and psychological well-being of older Chinese immigrants** | Liu, Jinyu; Mao, Weiyu; Guo, Man; Xu, Ling; Chi, Iris; Dong, Xinqi | 2021 | immigration, social support, bond, friendship, health, grief, well being | loss of friends, age, marital status, social connections, | loneliness, quality of life, | increased loneliness, variations in quality of life, targetd social support | baseline data from population-based epidemiological study, fgace to face home interviews, t-test and chi-square analyses, linear and logistic regression analyses, Patient Health Questionnaire | Liu, J., Mao, W., Guo, M., Xu, L., Chi, I., & Dong, X. (2021). Loss of friends and psychological well-being of older Chinese immigrants. *Aging & Mental Health*, 25 (2), 323–331. https://doi.org/10.1080/13607863.2019.1693967 | |
| **Kinship bereavement and psychological well-being of U. S. Chinese older women and men** | Lu, Peiyi; Shelley, Mack; Chen, Yiwei; Dong, Xinqi | 2022 | relationship, family, grief, loss, chinese, elderly, emotions | loss of kinship members, gender, culture | loneliness, stress, anxiety, health | increased loneliness, elevated stress levels, heightened anxiety, widow | face-to-face interviews, Patient Health Questionnaire-9, Perceived Stress Scale (PSS), Revised UCLA Loneliness Scale, Poisson regression, cross-sectional data from the Population Study of ChINese Elderly in Chicago Study (PINE) | Lu, P., Shelley, M., Chen, Y., & Dong, X. (2022). Kinship bereavement and psychological well-being of U.S. Chinese older women and men. *Journal of Women & Aging*, 34(1), 43–53. https://doi.org/10.1080/08952841.2020.1774226 | |
| **Meaning and experience of international migration in black african south african families** | Mabandla, Nthopele; Marchetti-Mercer, Maria C.; Human, Leonie | 2022 | International migration,<br>Black/South African Families<br>Transnationalism<br>Family Dynamics<br>Cultural Identity<br>Psychological Well being | Historical and Political context<br>Socioeconomic factors (aspiration for improved living)<br>cultural expectations and obligation | Transnational Family dynamics<br>Use of ICT-to facilitate communication and relationships<br>Emotional Ambivalence- mixed emotions | Redefinition of family roles<br>Psycholgocial impact - stress and anxiety<br>Economic Implications - dependencies | purposive and snowball sampling, interviews and referrals | Mabandla, N., Marchetti-Mercer, M. C., & Human, L. (2023). Meaning and Experience of International Migration in Black African South African Families. *Contemporary Family Therapy*, 45(4), 475–488. https://doi.org/10.1007/s10591-022-09651-8 | |
| **Tropical Cyclones and the Mobility of Older Persons: Insights from Coastal Bangladesh** | Malak, M. A.; Lina, N. K. | 2024 | tropical cyclones<br>older persons<br>relocate/evacuate<br>mobility<br>coastal Bangladesh<br>climate<br>disaster preparedness<br>vulnerability<br>resilience | Geographical exposure - high susceptibility<br>socioeconomic status - limited resources = hinder ability<br>health and physical limitations | vulnerability<br>chronic illness<br>dependence on social networks<br>access to information | **increased mortality and morbidity**<br>**displacement and loss of livelihood**<br>**psychological distress** | qualitatve appraoch, semi-structured interviews, focus groups, purposive sampling method | Scott, M., Ahmad, N., & Jolly, S. (2024). Tropical Cyclones and the Mobility of Older Persons: Insights from Coastal Bangladesh. *In Climate-Related Human Mobility in Asia and the Pacific*. Springer. | |
| **Older female Iraqi refugees in the United States: Voices of struggle and strength** | Nashwan, A.; Cummings, S. M.; Gagnon, K. | 2019 | Female iraqi refugess<br>older<br>resettlement<br>acculturation<br>resilience<br>conservation of resources theory<br>cultural adjustment<br>social support<br>stress | Pre-Migration Trauma<br>Cultural displacement<br>health challenges | Resilience<br>social support networks<br>cultural preservation | psychological well being<br>scoial integration<br>Positive health outcomes from effective coping mechanisms | audio-recorded interviews, phenomenological qualitative research methods | Nashwan, A., Cummings, S. M., & Gagnon, K. (2019). Older female Iraqi refugees in the United States: Voices of struggle and strength. *International Social Work*, 62(2), 653–668. https://doi.org/10.1177/0020872817742699 | |
| **Aging Muslim immigrants transitioning from Muslim majority countries to Muslim minority countries: A scoping review addressing dynamics of occupation, place, and identity** | Nasir, Nada; Hand, Carri; Rudman, Debbie Laliberte | 2024 | elder<br>muslim immigrants<br>occupation<br>place<br>identity<br>acculturation<br>cultural transition<br>social integration | Migration from Muslim Majority to Muslim Minority Countries<br>Cultural and Religious Practices<br>Established rituals<br>Aging related factors | Occupational Engagement<br>Sense of Place - connection and belonging<br>Identity Negotiation<br>purpose and identity | Acculturation stress<br>Social isolation or integration<br>Religious and cultural adaptation<br>Psychological well being or distress<br>Mixed emotions<br>intergenerational relationships | scoping review | Nasir, N., Hand, C., & Rudman, D. L. (2024). Aging Muslim immigrants transitioning from Muslim majority countries to Muslim minority countries: A scoping review addressing dynamics of occupation, place, and identity. *Journal of Occupational Science*, 31(3), 606–626. https://doi.org/10.1080/14427591.2023.2235368 | |
| **Working with Russian-Jewish immigrants in end-of-life care settings** | Newhouse, L. | 2013 | Russian-Jewish Immigrants<br>end of life<br>health<br>cultural sensitvity<br>historical trauma<br>communication barriers<br>palliative care<br>grief/loss | Historical and cultural background<br>religious suppression<br>Previous traumatic experiences<br>anti-Semitism<br>Communication styles<br>Stigma of death/dying conversation | Reluctance to discuss end of life<br>Preference for family centered decision making<br>Skepticism towards Palliative Care<br>Cultural barriers | Underutilization of hospice services<br>Increased stress for families/caregiver burnout<br>Potential for miscommunication | Case vignettes - a Russian-Jewish immigrant and a professional social worker, draws on his clinical experience through 2 decades | Newhouse, L. (2013). Working with Russian-Jewish Immigrants in End-of-Life Care Settings. *Journal of Social Work in End-of-Life & Palliative Care*, 9(4), 331–342. https://doi.org/10.1080/15524256.2013.846884 | |
| **Voices Unheard: A Reflective Lifeworld Research Study of Older Arabic-Speaking Female Migrants and Their Experience of Existential Loneliness** | Olofsson, J.; Sjögren-Forss, K.; Bramhagen, A. C.; Rämgård, M. | 2024 | loss<br>existential loneliness<br>communication<br>elders<br>Arabic speaking female migrants<br>Reflective Lifeword Research<br>Migration<br>Cultural and religious coping<br>traditional beliefs | Migration and displacement<br>Loss of social networks<br>cultural and language barriers<br>Aging and health decline | Sense of isolation<br>Search for meaning<br>religious and spiritual engagement<br>mixed emotions | Diminished sense of mental and emotional wellbeing<br>Social withdrawal<br>Reliance on religious communities<br>impact on healthcare engagement | phenomenological approach, interviews | Olofsson, J., Sjögren-Forss, K., Bramhagen, A., & Rämgård, M. (2024). Voices Unheard: A Reflective Lifeworld Research Study of Older Arabic-Speaking Female Migrants and Their Experience of Existential Loneliness. International Journal of Older People Nursing, 19(4), e12633-n/a. https://doi.org/10.1111/opn.12633 | |
| **If i had stayed back home, i would not be alive any more⋯ - Exploring end-of-life preferences in patients with migration background** | Paal, P.; Bükki, J. | 2017 | end of life preference<br>health<br>migration<br>economics of migration<br>palliative care<br>cultural sensitivity<br>healthcare access | Migration History - adverse conditions<br>Healthcare system navigation<br>cultural and religious beliefs | Preference for Family involvement<br>Desire for clear communication<br>Consideration of returning to country of origin due to familiarity | Potential for misaligned care<br>underutilization of palliative services<br>emotional distress | Two-armed study using Kaufmann's 'understanding interview' ('focused interview')<br>grounded theory approach. | Paal, P., & Buekki, J. (2017). "If I had stayed back home, I would not be alive any more..." - Exploring end-of-life preferences in patients with migration background. *PloS One*, 12(4), e0175314–e0175314. https://doi.org/10.1371/journal.pone.0175314 | |

| Article Name | Authors | Year of Publication | Relevant Concepts (i.e., keywords, key concepts discussed) | Antecedents: Events or situations that need to happen before the concept can occur.<br>What is required for the concept to occur? (Factors that affect, stimulate or encourage the concept to occur) | Defining Attributes: Precise terms that can be used to clarify a concept, aiding in differentiating it from other similar concepts | Consequences: The events or situations that may arise following the manifestation of a concept, frequently inspiring fresh perspectives or research paths related to a specific concept. (The outcomes as a result of the concept) | Empirical Referents: Are measurable occurrences of the phenomenon that facilitate the concept's quantification. (i.e. the name of theory, framework, scale for measurement, survey) | Citation (APA 7th Edition) | Notes |
|---|---|---|---|---|---|---|---|---|---|
| **Addressing Hearing Loss of Palestinians Living in Refugee Camps** | Pakulski, Lori A. | 2024 | unaddressed or inadequately managed hearing loss<br>palestinian refugee camps<br>culturally sensitive interventions<br>community based hearing healthcare<br>humanitarian audiology services | Prolonged displacement<br>limited access to healthcare<br>Cultural perceptions | community engagement<br>capacity building<br>humanitarian outreach | improved quality of life<br>educational advancement<br>improved social participation<br>economic empowerment<br>strengthened healthcare infrastructure | Article discussion | Pakulski, L. A. (2024). Addressing Hearing Loss of Palestinians Living in Refugee Camps. *Perspectives of the ASHA Special Interest Groups*, 9(4), 1188–1196. https://doi.org/10.1044/2024_PERSP-23-00251 | |
| **Soy diferente: A qualitative study on the perceptions of recovery following traumatic brain injury among Spanish-speaking US immigrants** | Pappadis, Monique R.; Sander, Angelle M.; Struchen, Margaret A.; Kurtz, Diana M. | 2022 | traumatic brain injusry<br>quality of life<br>self-concept<br>spanish speaking<br>Hispanic Immigrants America<br>Cultural perceptions<br>recovery | cultural background-impact view on health<br>poor healthcare accessibility-umfamiliarity<br>level of social support system | faith and hopefulness<br>empathy and altruism<br>perception of self | enahnced community integration<br>employment and financial stability<br>psychological wellbeing<br>stigma reduction | Semi-structured interviews | Pappadis, M. R., Sander, A. M., Struchen, M. A., & Kurtz, D. M. (2022). Soy diferente: a qualitative study on the perceptions of recovery following traumatic brain injury among Spanish-speaking U.S. immigrants. *Disability and Rehabilitation*, 44(11), 2400–2409. https://doi.org/10.1080/09638288.2020.1836045 | |
| **Paradise lost: Older Cuban American exiles' ambiguous loss of leaving the homeland** | Perez, Rose M. | 2013 | families<br>loss/grief<br>ambiguous loss<br>Cuban American<br>Exile/displacement<br>psychological impact<br>Cuban revolution/diaspora | Historical context of Cuban Revolution<br>Perception of temporary exile<br>Uncertainty regarding the homeland | feelings of betrayal<br>expectation of temporary refuge<br>longing and idealization of the past/homeland | chronic ambiguous loss<br>identity and relationship challenges<br>Cultural preservation efforts | transcendental phenomenology<br>Composites of participants' textural and structural descriptions of phenomena<br>Stevick-Colaizzi-Keen method- to find common themes<br>writing and audio recording field notes | Perez, R. M. (2013). Paradise Lost: Older Cuban American Exiles' Ambiguous Loss of Leaving the Homeland. *Journal of Gerontological Social Work*, 56(7), 596–622. https://doi.org/10.1080/01634372.2013.817496 | |
| **Getting old in Little Lhasa: Experiences of aging in Dharamsala** | Rajesh, M. N. | 2018 | aging<br>Tibetan Refugees<br>Dharamsala<br>Cultural identity preservation<br>exile and displacment<br>intergenerational relationships | historical displacements<br>expectation of return<br>Cultural assimilation practices | community cohesion<br>cultural transmission<br>resilience amidst uncertainty | enahnced social support<br>cultural enrichment<br>psychological well-being | chapter in a book | Rajesh, M. (2018). Getting Old in Little Lhasa: Experiences of Aging in Dharamsala. *In Handbook of Research on Multicultural Perspectives on Gender and Aging* (pp. 177–189). | |
| **Associations between memory loss and trauma in US asylum seekers: A retrospective review of medico-legal affidavits** | Saadi, A.; Hampton, K.; de Assis, M. V.; Mishori, R.; Habbach, H.; Haar, R. J. | 2021 | trauma<br>memory loss<br>asylum seekers<br>medico-legal affidavits<br>neuropsychiatric symptoms<br>displacement | exposure to trauma<br>migration and displacment<br>legal proceedings | memory impairments<br>neuropsychiatric symptoms<br>legal documentation | challenges in legal proceedings (from memory impairments)<br>mental health implications<br>diminished quality of life<br>needs for culturally specialized support - legal and health | random selection of 200 medico-legal affidavits, and data was extracted using a coding manual<br>multiple logistic regression<br>qualitative content analysis of the affidavits | Saadi, A., Hampton, K., de Assis, M. V., Mishori, R., Habbach, H., & Haar, R. J. (2021). Associations between memory loss and trauma in US asylum seekers: A retrospective review of medico-legal affidavits. *PloS One*, 16(3), e0247033-. https://doi.org/10.1371/journal.pone.0247033 | |
| **Bereavement and meaning reconstruction among Japanese immigrant widows: Living with grief in a place of marginality and liminality in the United States** | Saito, Chizuko | 2014 | bereavement<br>meaning reconstruction<br>Japanese Immigrant Widows<br>Marginality and liminality<br>grief/social exclusion<br>spiritual and cultural support | migration and cultural transition<br>international marriages<br>social and cultural marginalization | reconstructing life post-loss (after spouse death)<br>acceptance of human finitude<br>maintaining bond with the deceased- rituals and emotional connections<br>establishing communities | enhanced spiritual and cultrual connections<br>psychological adaptations<br>strengthened community bonds | ethnographic study<br>autobiographical approachs<br>emi-structured ethnographic interviews<br>participant observation<br>tape-recorded interviews | Saito, C. (2014). Bereavement and Meaning Reconstruction among Japanese Immigrant Widows: Living with Grief in a Place of Marginality and Liminality in the United States. *Pastoral Psychology*, 63(1), 39–55. https://doi.org/10.1007/s11089-013-0517-9 | |
| **A place to grow older … alone? Living and ageing as a single older lifestyle migrant in the Azores** | Sampaio, D. | 2018 | single older lifestyle migrants<br>aging<br>Azores<br>Place attachment<br>life narratives | migration for lifestyle choices<br>loss of spouse<br>retirement and mobility | individual agency in relocation<br>complex relationship with place<br>evolving social connections | enhanced self-actualization<br>ambivalent social integration<br>reconfiguration of identity | in-depth life narrative interviews<br>diverse selection, snowballing<br>"free-flowing" approach, letting the interviewees guide their own narratives<br>comparative analysis<br>fieldnotes, participant observation, photographs | Sampaio, D. (2018). A place to grow older … alone? Living and ageing as a single older lifestyle migrant in the Azores. *Area (London 1969), 50*(4), 459–466. https://doi.org/10.1111/area.12414 | |
| **Feeling at home: Korean americans in senior public housing** | Seo, Yoon Kyoung; Mazumdar, Sanjoy | 2011 | Korean American Seniors<br>Senior Public Housing<br>Sense of Home<br>Belonging<br>Cultural Identity/adaptation<br>Social Integration<br>relocation decision making | immigration and settlement<br>family dynamics<br>cultural expectation | adaptation to new living spaces<br>negotiation of cultural identity<br>developemnt of social networks | enhanced social support<br>strengthened cultural connections<br>imporved quality of life | naturalistic field research- close up examination of physcial setting<br>ethnography<br>observation<br>open-ended interviews<br>field notes, sketches, and photographs | Seo, Y. K., & Mazumdar, S. (2011). Feeling at home: Korean Americans in senior public housing. *Journal of Aging Studies, 25*(3), 233–242. https://doi.org/10.1016/j.jaging.2011.03.008 | |
| **Age Melancholy of Older Mizrahi Women Residing in Tel Aviv as a Social Loss: Exploring Intersections of Health and Social Support in an Ethnographic Study** | Shamur, T. | 2025 | Age melancholy<br>older Mizrahi Women<br>Social Loss<br>health and social support<br>lived experiences | Empty Nest Syndrome<br>Cultural social displacement<br>economic challenges | Loss of social engagements<br>intergeneratonal disconnect<br>poor health implications | strengthened social supoort networks<br>imporved health outcomes<br>cultural preservation | participant visits<br>weekly focus groups<br>recording<br>thematic analysis method<br>ethnographic research<br>participant observation | Shamur, T. (2025). Age Melancholy of Older Mizrahi Women Residing in Tel Aviv as a Social Loss: Exploring Intersections of Health and Social Support in an Ethnographic Study. *Qualitative Health Research, 35*(1), 31–43. https://doi.org/10.1177/10497323241263238 | |

| **Article Name** | **Authors** | **Year of Publication** | **Relevant Concepts** (i.e., keywords, key concepts discussed) | **Antecedents:** Events or situations that need to happen before the concept can occur.<br>What is required for the concept to occur? (Factors that affect, stimulate or encourage the concept to occur) | **Defining Attributes:** Precise terms that can be used to clarify a concept, aiding in differentiating it from other similar concepts | **Consequences:** The events or situations that may arise following the manifestation of a concept, frequently inspiring fresh perspectives or research paths related to a specific concept. (The outcomes as a result of the concept) | **Empirical Referents:** Are measurable occurrences of the phenomenon that facilitate the concept's quantification. (i.e. the name of theory, framework, scale for measurement, survey) | **Citation (APA 7th Edition)** | **Notes** |
|---|---|---|---|---|---|---|---|---|---|
| **"My Last Husband and Marriage:" The Impact of Inheritance Disputes on Chinese Immigrants' Widowhood in the United States** | Shi, Hua | 2022 | -Spousal bereavement<br>-Lived experiences<br>-Immigration<br>-Immigrant older adult<br>-Transnational marriage<br>-Inheritance disputes<br>-Cultural conflicts<br>-Blended families | - Migration to U.S. for marriage<br>-Stepfamily dynamics<br>-Legal vulnerability<br>-Finanicial vulnerability<br>-Cultural expectations of widowhood | - Emotional toll related to disinheritence feelings of betrayal and injustice in inheritance disputes<br>- Social reprecussions, loss of friendships and estrangement from social circles<br>- Financial Hardship due to economic dependence on late spouses and loss of financial stability | -Increased emotional isolation through widowhood<br>-Legal battles over inheritence<br>-Personal growth and independence<br>-Resiliency<br>-Need for legal + emotional support services through bilingual legal aid and bereavement counselling | - cross-cultural communication theory | Shi, H. (2022). “My last husband and marriage:” The impact of inheritance disputes on Chinese immigrants’ widowhood in the United States. *Ageing International*, *47*(4), 653-671. | |
| **Health status and health needs of older refugees from Syria in Lebanon** | Strong, J.; Varady, C.; Chahda, N.; Doocy, S.; Burnham, G. | 2015 | - Refugees<br>-Syrians<br>-Older populations<br>-Disabilities<br>-Non-communicable diseases<br>-Lebanon<br>-Aging<br>- Healthcare access<br>-Social support + dependency | - Conflict and forced displacement from Syria<br>- Lack of access to stable income, housing, and social support<br>- Barriers to healthcare<br>-Cost and limited availability of medical supplies<br>-Psychological trauma<br>-Post-migration stress | - High prevalence of chronic diseases like hypertension, diabetes, heart disease<br>- Physical disabilities limiting mobility and independence<br>- High dependency on humanitarian assistance for food and healthcare<br>-Increased emotional distress due to separation from family and loss of social status | - Increased rates of malnutrition due to food insecurity<br>- Social isolation<br>-Heightened feelings of being a burden to families<br>- Limited access to medical care<br>- Worsening health conditions<br>- Overlooked needs of elderly refugees in humanitarian responses focusing primarily on women and children | - Used the a systematic selection of refugees where Caritas Lebanon Migrant Center (CLMC) and Palestinian Women’s<br>Humanitarian Organization (PALWHO social workers collected qualitative and quantitative info. | Strong, J., Varady, C., Chahda, N., Doocy, S., & Burnham, G. (2015). Health status and health needs of older refugees from Syria in Lebanon. *Conflict and Health* , *9*, Article 12. https://doi.org/10.1186/s13031-014-0029-y | |
| **Impact of war and resettlement on Vietnamese families facing dementia: A qualitative study** | Sun, Mengxue; Tran, Duyen; Bach, Anna; Ngo, Uyen; Tran, Tiffany; Do, Thuy; Meyer, Oanh L. | 2022 | -Dementia<br>-Caregiving<br>-Qualitative analysis<br>-Trauma<br>-Asian<br>-Refugee issues<br>-Migration + resettlement issues<br>- Lack of culturally tailored healthcare support | -Pre-migration trauma due to war<br>-Forced resettlement<br>-Separation from family members<br>-Psychological distress affecting both caregivers and patients<br>-Language barriers<br>-Limited access to healthcare resources | -Intersections of war trauma<br>-Dementia progression<br>-Caregiver burden<br>-Cultural stigma around dementia<br>-Role of filial piety in caregiving expectations<br>-Social isolation<br>-Lack of systemic support | -Increased rates of stress related health problems among caregivers<br>-Greater likelihood of undiagnosed/ untreated dementia<br>-Higher prevalence of depression + anxiety in both patients and caregivers<br>-Calls for culturally sensitive mental health interventions | -Family Caregiver Alliance framework for assessing caregiver burden<br>-In-person interviews with a descriptive analysis approach | Sun, M., Tran, D., Bach, A., Ngo, U., Tran, T., Do, T., & Meyer, O. L. (2022). Impact of War and Resettlement on Vietnamese Families Facing Dementia: A Qualitative Study. *Clinical Gerontologist*, *45*(4), 798-807. https://doi.org/10.1080/07317115.2022.2071661 | |
| **Settlement in Nanjing among Chinese rural migrant families: The role of changing and persistent family norms** | Tang, S.; Zhou, J.; Druta, O.; Li, X. | 2023 | - China<br>-Family norms<br>-Inter-generational relation<br>-Migrant<br>-Settlement<br>-Younger people<br>-Rural-to-urban migration<br>-Hukou system<br>-Housing affordability<br>-Employment security<br>-Gender roles | - Hukou system restrictions limiting migrants' access to urban welfare services<br>- Economic disadvantages<br>- High housing costs in urban areas<br>- Family expectations regarding parental support for adult children's settlement<br>- Declining filial obligation among younger generations | - Persistent intergenerational dependence for financial + social support<br>- Gender norms shifting toward greater equality<br>- parental sacrifice remaining crucial from a cultural perspective<br>-Younger migrants prioritizing self-fulfillment over traditional family obligations | -Economic burden shifting from young migrants to their elderly parents<br>-Increased pressure on family relations<br>-Increased pressure on intergenerational negotiations<br>- Social inequalities highlighted by institutional barriers (hukou system, limited welfare access)<br>- Gradual erosion of traditional values<br>-Favor of individualistic aspirations | - New Economics of Labor Migration (NELM) framework<br>-Qualitative data analysis using NVIVO software | Tang, S., Zhou, J., Druta, O., & Li, X. (2023). Settlement in Nanjing among Chinese rural migrant families: The role of changing and persistent family norms. *Urban Studies*, *60*(6), 1083-1101. https://doi.org/10.1177/00420980221130761 | |

| Article Name | Authors | Year of Publication | **Relevant Concepts** (i.e., keywords, key concepts discussed) | **Antecedents:** Events or situations that need to happen before the concept can occur.<br>What is required for the concept to occur? (Factors that affect, stimulate or encourage the concept to occur) | **Defining Attributes:** Precise terms that can be used to clarify a concept, aiding in differentiating it from other similar concepts | **Consequences:** The events or situations that may arise following the manifestation of a concept, frequently inspiring fresh perspectives or research paths related to a specific concept. (The outcomes as a result of the concept) | **Empirical Referents:** Are measurable occurrences of the phenomenon that facilitate the concept's quantification. (i.e. the name of theory, framework, scale for measurement, survey) | Citation (APA 7th Edition) | Notes |
|---|---|---|---|---|---|---|---|---|---|
| **Loneliness experiences of Hmong older adults: A constructivist grounded theory study** | Vang, Cindy | 2019 | - Loneliness<br><br>- Social isolation<br><br>-Cultural identity<br><br>- Intersectionality<br><br>-Migration<br><br>-Displacement<br><br>-Refugee experience<br><br>-Trauma<br><br>-Coping mechanisms<br><br>-Instability | -War, forced migration, displacement, and cultural disruption<br><br>-Separation from family members<br><br>-Loss of social roles<br><br>-Language barriers<br><br>-Limited access to culturally appropriate mental health services.<br><br>- Gender norms affecting social status and expectations<br><br>- Limited economic opportunities<br><br>- Limited social integration | - Loneliness conceptualized as both physical and emotional distress<br><br>- Situational loneliness (temporary) and chronic loneliness (lifelong) as loneliness intensities.<br><br>- Influence of cultural norms in defining acceptable emotional expressions and coping mechanism | - Psychological distress, depression and suicidal ideation<br><br>- Internalized stigma of being an outsider, further emphasising loneliness<br><br>-Limited access to support systems and social services<br><br>- Adaptation strategies such as community-based support groups, reliance on family, or religious practices | - Peplau and Perlman's cognitive discrepancy theory<br><br>- Crenshaw's intersectionality theory<br><br>- Weiss' Interactionist theory<br><br>-Intersectionality critique of loneliness theories<br><br>-Atlas.ti software for coding and analyzing qualitative data | Vang, C. (2019). *Loneliness experiences of Hmong older adults: A constructivist grounded theory study* [Doctoral dissertation, Arizona State University]. ASU Electronis Theses and Dissertations. https://keep.lib.asu.edu/items/157586 | |
| **Conceptualizing loneliness among a Hmong older adult group: Using an intersectionality framework** | Vang, Cindy; Sieng, Michael; Zheng, Mingyang | 2023 | - Refugees<br><br>-Mental health<br><br>-Migration<br><br>-Grounded theory<br><br>-Intersectionality<br><br>- Loneliness<br><br>-Cultural marginalization<br><br>- Identity | - War-related trauma<br><br>-Forced migration<br><br>- Language barriers preventing access to social integration and mental health services<br><br>- Cultural stigma around expressing loneliness or seeking mental health support<br><br>-Structural inequalities | - Loneliness as an evolving experience influenced by migration phases<br><br>- Interconnection between identity, cultural values, and social support<br><br>- Intersectionality affecting experiences differently based on gender, marital status, and social position | -Lack of access to social integration and mental health services<br><br>- Social disconnection<br><br>- Limitation of Hmong older adults' access to resources and community networks<br><br>-Physical (headaches, insomnia) and emotional (sadness, depression) deteriorations as an outcome of chronic loneliness<br><br>-Increased reliance on spiritual and cultural coping strategies<br><br>-Social isolation resulting in a diminished sense of belonging<br><br>- Calls for culturally + linguistically responsive actions to support aging refugees | - Grounded theory analysis used for data collection<br><br>-Crenshaw's intersectionality framework | Vang, C., Sieng, M., & Zheng, M. (2023). Conceptualizing loneliness among a Hmong older adult group: Using an intersectionality framework. *Asian American Journal of Psychology*, *14*(4), 340-349. https://doi.org/10.1037/aap0000307 | |
| **Influencing Factors of Loneliness Among Hmong Older Adults in the Premigration, Displacement, and Postmigration Phases** | Vang, C.; Thor, P.; Sieng, M. | 2021 | - Aging<br><br>- Southeast Asian<br><br>-Grounded theory<br><br>-Loneliness<br><br>-Intersectionality | -Betrayal + war related violence during premigration<br><br>- Familial loss<br><br>-Instability<br><br>- Language barriers in postmigration<br><br>-Cultural adaptation challenges in postmigration<br><br>-Loss of social status<br><br>-Diminished filial piety specific to gender | - Loneliness as an intersectional experience shaped by historical trauma<br><br>- Social identity shifts across premigration, displacement, and postmigration<br><br>- Compounded experiences of discrimination + exclusion<br><br>- Gendered familial influences on loneliness experiences | -Increased prevalence of anxiety, PTSD, and depression among older Hmong refugees<br><br>- Intergenerational conflicts<br><br>-Shifting family roles in host countries<br><br>- Economic disadvantages<br><br>-Social isolation<br><br>- Calls for policy interventions + culturally sensitive mental health support | - Crenshaw's intersectionality framework<br><br>- Constructivist grounded theory study | Vang, C., Thor, P., & Sieng, M. (2021). Influencing factors of loneliness among Hmong older adults in the premigration, displacement, and postmigration phases. *Journal of Refugee Studies*, *34*(3), 3464-3485. https://doi.org/10.1093/jrs/feab029 | |
| **The lived experiences of spousal bereavement and adjustment among older Chinese immigrants in Calgary** | Wang, Qianyun; Walsh, Christine A.; Tong, Hongmei | 2023 | - Spousal bereavement<br><br>-Lived experiences<br><br>-Immigration<br><br>-Chinese immigrant<br><br>-Older adults<br><br>-Cultural beliefs about death and mourning<br><br>-Ethno-cultural communities | - Cultural and religious perceptions of death (Confucianism, Taoism, Buddhism)<br><br>-Immigrant status affecting social networks+ access to grief support<br><br>-Economic dependency on children post-migration<br><br>- Emotional dependency on children post-migration<br><br>-Reluctance to seek professional mental health services | -Prolonged and private grieving process<br><br>-Reliance on cultural + religious rituals for coping<br><br>-Minimal external bereavement support from formal institutions<br><br>-Preference for familial and community-based assistance over professional help | -Increased social withdrawal<br><br>-Increased loneliness<br><br>-Reduced access to mental health care due to cultural stigma<br><br>- Limited adaptation to widowhood compared to non-immigrant populations<br><br>-Potential for intergenerational conflict regarding grief practices | - Descriptive phenomenology as a theoretical lens<br><br>-Windle and Bennettecological resilience framework<br><br>-Model of continuing bond | Wang, Q., Walsh, C.A., & Tong, H. (2023). The lived experiences of spousal bereavement and adjustment among older Chinese immigrants in Calgary. *Journal of Cross-Cultural Gerontology, 38* (2), 137-154. https://doi.org/10.1007/s10823-023-09477-3 | |

| Article Name | Authors | Year of Publication | **Relevant Concepts** (i.e., keywords, key concepts discussed) | **Antecedents:** Events or situations that need to happen before the concept can occur.<br>What is required for the concept to occur? (Factors that affect, stimulate or encourage the concept to occur) | **Defining Attributes:** Precise terms that can be used to clarify a concept, aiding in differentiating it from other similar concepts | **Consequences:** The events or situations that may arise following the manifestation of a concept, frequently inspiring fresh perspectives or research paths related to a specific concept. (The outcomes as a result of the concept) | **Empirical Referents:** Are measurable occurrences of the phenomenon that facilitate the concept's quantification. (i.e. the name of theory, framework, scale for measurement, survey) | Citation (APA 7th Edition) | Notes |
|---|---|---|---|---|---|---|---|---|---|
| **We were meant to go down one road, but now we have rerouted': A phenomenological inquiry into the experience of aging out-of-place** | Wijekoon, Sachindri | 2018 | -Aging out of place<br><br>-Late life immigration<br><br>-Occupational engagement<br><br>-Habit reconfiguration<br><br>-Transactional perspective<br><br>-Human occupation<br><br>-Phenomenology<br><br>-Transactionalism<br><br>-Occupational science | -Immigration in old age<br><br>-Loss of familiar social and cultural structures<br><br>-Displacement from familiar environments and roles<br><br>-Need for adaptation due to cultural, linguistic, and social differences<br><br>-Lack of extended social support in host country | -Continuous renegotiation of daily life<br><br>-Reconstructing a sense of belonging through new habits and roles<br><br>-Selective acculturation and identity negotiation<br><br>-Emotional and social dissonance between native and host country expectations | -Development of cultural habits<br><br>-Development of occupational habits<br><br>-Psychological stress due to role reversal<br><br>-Altered life trajectory compared to expectations<br><br>-Positive adaptation through cultural integration and habit transformation<br><br>-Qualitative phenomenological methods for analyzing lived experiences | - John Dewey's Transactional Theory<br><br>-Western conceptual frameworks<br><br>- Max van Manen's framework for phenomenological analysis<br><br>-Wilcock's occupational perspective of health<br><br>-Transactional perspective on occupation<br><br>-Interpretive phenomenological analysis (IPA)<br><br>-de Witt & Ploeg's framework of expressions | Wijekoon, S. (2018). *'We were meant to go down one road, but now we have rerouted': A phenomenological inquiry into the experience of aging out-of-place* (Publication No. 5569) [Doctoral thesis, The University of Western Ontario]. Electronic Thesis and Dissertation Repository. https://ir.lib.uwo.ca/etd/5569 | GO OVER* |
| **Ageing in the bush: The role of rural places in maintaining identity for long term rural residents and retirement migrants in north-east Victoria, Australia** | Winterton, R.; Warburton, J. | 2012 | - Rural ageing<br><br>- Identity<br><br>- Retirement migration | - Population aging trends in rural areas<br><br>- Rural urban migration patterns affecting community demographics<br><br>- Economic + social factors influencing the decision to age in place or migrate for retirement<br><br>-Health + mobility changes limiting interaction with place | - "placelessness"<br><br>- Experience variation of long term rural residents + retirement migrants<br><br>-Community participation<br><br>- Psychological and social benefits<br><br>-Continuity in place identity | - outmigration of younger people<br><br>-Risk of isolation if community support structures decline<br><br>- Social cohesion<br><br>- Wellbeing benefit<br><br>- Strengthened identity<br><br>-Challenges in accessing healthcare + services in aging, rural regions | - Concepts of "Place-identity", "sense of community", "place dependence", "place attachment"<br>- "Breakwell's (1986, 1992) identity process theory to examine the influence of place, or facets of place, on personal identity"<br>- Additional concepts discussed: distinctiveness, place referent, place-congruent continuity, self-esteem, self-efficacy, sense of community, place dependence, place attachment | Winterton, R., & Warburton, J. (2012). Ageing in the bush: The role of rural places in maintaining identity for long term rural residents and retirement migrants in north-east Victoria, Australia. *Journal of Rural Studies*, *28* (4), 329-337. https://doi.org/10.1016/j.jrurstud.2012.01.005 | |
| **Culturally and linguistically diverse older adults relocating to residential aged care** | Yeboah, Cecilia; Bowers, Barbara; Rolls, Colleen | 2013 | relocation, older adults, pathways, Australian, residential aged care, culturally and linguistically diverse residents | - relocation to nursing homes | Preceding losses before relocation to nursing home<br>- Physical losses, including loss of ability to perform activities of daily living due to factors related to pain, endurance, balance, and mobility<br>- Relational losses, including death of spouse, losing important relationships<br>- Support losses, including functional support from loved ones, support person, and caregiver (due to respite, illness, divorce, relocation, disapproval from health professional) | Attempt to reverse or replace losses by:<br>- Increased healthseeking behaviours to improve health and mobility, such as attending rehab<br>- Seeking out informal support services (e.g. meals on wheels) | - interviews using grounded theory approach<br>- "Pathway to nursing home relocation" | Yeboah, C., Bowers, B., & Rolls, C. (2013). Culturally and linguistically diverse older adults relocating to residential aged care. *Contemporary Nurse*, *44*(1), 50-61. https://doi.org/10.5172/conu.2013.44.1.50 | |
| **Mass Relocation and Depression Among Seniors in China** | Zeng, W.; Wu, Z.; Schimmele, C. M.; Li, S. | 2015 | China, depression, elderly individuals, mental health, migration | - Relocation to conserve ecological spaces | - Loss of social support | - Higher rates of depression, increased stress | Stress process model (to study state-organized relocation (mass internal migration) and depression);<br>- 9-item version of the Center for Epidemiologic Studies–Depression (CES-D)<br>Scale<br>- life-course perspective in relation to relocation (relocation as an off-time event causing stress) | Zeng, W., Wu, Z., Schimmele, C. M., & Li, S. (2015). Mass relocation and depression among seniors in China. *Research on Aging*, *37*(7), 695-718. https://doi-org.ezproxy.lib.torontomu.ca/10.1177/0164027514551178 | |
| **The making of ageing migrant masculinities: loss and recuperation in the lived experiences of Chinese and Korean older adult migrant men** | Zhu, S.; Chan, D. | 2024 | Ageing; masculinity; migration; older migrants; gender identity | - Migration and aging | - Sense of loss<br>- Loss of traditional male gender roles, social capital, status<br>- Loss of physical ability<br>- Lack self-worth<br>- Loss of healthy body = loss of masculinity | - Anxiety, decreased self-worth, feelings of shame<br>- Adoption of new social roles and responsibilities, such as volunteering and child-care, educator for new generations; new leadership roles<br>- "a reassertion of patriarchal power in the family as head of the household, and body work as a form of individual agency."<br>- reinterpretation of nontraditional/female roles as a form of "masculine duty and responsibility", "validates ... sense of usefulness and worth as male leaders in the family" | "'embodied perspective'"; concept of "migrant-body"; "double-jeorpary" or double marginalization | Zhu, S., & Chan, D. (2024). The making of ageing migrant masculinities: loss and recuperation in the lived experiences of Chinese and Korean older adult migrant men. *Journal of Ethnic and Migration Studies*, 1–16. https://doi.org/10.1080/1369183X.2024.2305683 | ** GO OVER |
